# Developmental and adult expression of the Meis2 transcription factor in the central nervous system of *Xenopus laevis*: a developmental and evolutive analysis

**DOI:** 10.3389/fnana.2025.1677413

**Published:** 2025-11-06

**Authors:** Ruth Morona, Ana Martinez, Nerea Moreno

**Affiliations:** Departamento de Biología Celular e Histología, Facultad de Biología, Universidad Complutense de Madrid, Madrid, Spain

**Keywords:** pallium, amygdala, hypothalamus, optic tectum, rhombencephalon, patterning, brain evolution

## Abstract

Myeloid ecotropic viral integration site 2 (Meis2) is a three-amino-acid-loop-extension (TALE) transcription factor (TF) involved in key neurodevelopmental processes, such as neuronal differentiation and brain regionalization. Its expression is well documented in amniotes and teleosts, but its distribution in the developing brain of anamniote tetrapods remains poorly understood. Therefore, the distribution of Meis2-immunoreactive (-ir) cells was analyzed throughout the developmental stages of the *Xenopus laevis* brain, revealing a dynamic, stage-specific expression pattern. From the early embryonic stages, Meis2-ir cells were found in the telencephalon, specifically in the ventrolateral pallium and subpallium; the diencephalon, particularly in the prosomere 3 and transiently in p2 and in the habenula; the optic tectum; the mesencephalic tegmentum; and the rhombencephalon. During the premetamorphic stages, Meis2 expression extended rostrally in the olfactory bulb (OB) and to subpallial derivatives, including scattered cells in the amygdaloid region. It was present in the alar and basal hypothalamus. During the metamorphic climax and juvenile phases, Meis2-ir expression became clearly defined in specific mature nuclei, specifically in the ventral pallium, the bed nucleus of the stria terminalis, septal nuclei, supra-paraventricular and mammillary hypothalamus, and prethalamic nuclei. In addition, from the metamorphic climax stages, Meis2 occupied a number of tectal layers and was observed in the cerebellar nucleus. The most prominent and constant expression was observed in the rhombencephalon, particularly in areas surrounding the isthmus and the reticular formation. This expression extended from rostral rhombomeres (r1–r3) caudally into the lateral line system and raphe nuclei. These results highlight the conserved and temporally regulated role of Meis2 in the regional specification and maturation of the central nervous system and reveal particularities related to cell specification.

## 1 Introduction

Myeloid ecotropic viral integration site (Meis) genes belong to the three-amino-acid-loop-extension (TALE) subfamily of transcription factors (TFs), which possess an atypical homeodomain characterized by a specific insertion of three amino acids (proline-tyrosine-proline, PYP) between the first and second helices of the HD. This insertion alters its geometry and improves both its ability to interact with other transcription factors and its DNA-binding specificity (Schulte, 2014).

In vertebrates, the Meis family includes Prep1, Prep2, Meis1, Meis2, and Meis3, with orthologs found in other organisms, such as homothorax in *Drosophila* and unc-62 in *Caenorhabditis elegans*. The homeodomain proteins of the TALE class are characterized by a DNA-binding domain of approximately 60 amino acids consisting of three α-helices where the third helix is the main DNA contact region (Ferretti et al., 2000). The insertion of the three amino acids between helices one and two allows interaction with other proteins and additional transcription factors, including members of the Hox and Pbx families. These complexes are critical for the regulation of gene activation or repression during embryonic developmental processes (Berthelsen et al., 1998; Ferretti et al., 2000).

These proteins often act as cofactors of the Hox and Pax genes and form multimeric complexes that regulate gene expression during key processes of embryonic development, such as body axis specification, neurogenesis, and cell differentiation (Longobardi et al., 2014; Moens and Selleri, 2006). Indeed, TALE factors, including Meis proteins, are involved in the creation of chromatin-permissive platforms selected by TFs whose expression is restricted to specific tissues. This role is critical because factors such as Hox often require prior exposure to TALE factors to gain access to their functional binding sites. This shows that TALE factors are not mere cofactors but central elements of tissue- and cell lineage-specific transcriptional regulation (Bobola and Sagerstrom, 2024). Although they were originally associated with Hox-dependent functions in the hindbrain, their involvement in mechanisms independent of these genes has also been demonstrated, particularly in the development of the anterior central nervous system (Agoston and Schulte, 2009; Heine et al., 2008).

In particular, myeloid ecotropic viral integration site 2 (Meis2), also known as Mrg1, encodes a transcription factor of approximately 410 amino acids that belongs to the Meis class (Berthelsen et al., 1998; Jacobs et al., 1999). The importance of Meis2 in neurogenesis has been demonstrated particularly through its cooperation with Pax6 in the differentiation of dopaminergic neurons in the olfactory bulb (OB) and retina and the proliferation of neural progenitor cells in the forebrain (Agoston et al., 2012, 2014). These functions are related to its ability to recruit co-activators and shift transcriptional repressors, thereby dynamically modulating gene expression (Choe et al., 2009, 2014). Meis2 is involved in the formation of the forebrain and neuroanatomical boundaries. Its expression in the early stages of telencephalon and diencephalon development suggests a key role in the regionalization of specific brain regions (Toresson et al., 2000; Ferran et al., 2007; Takahashi et al., 2008). In the hindbrain, it regulates the development of rhombomeres that are essential for the organization of cranial nerve nuclei and sensorimotor coordination (Ferretti et al., 2000; Wassef et al., 2008; Agoston and Schulte, 2009; Vitobello et al., 2011). In addition, mutations in the Meis2 gene have been associated with intellectual disability, cardiac defects, and a characteristic facial phenotype in humans (Giliberti et al., 2020). Meis2 is also essential for the survival and proliferation of neuroblastoma cells, as it transcriptionally regulates the progression of the M phase of the cell cycle (Zha et al., 2014). Therefore, Meis2 proves to be a key regulator in cell fate specification, neuronal maturation, and cell homeostasis.

The study of the brain developmental expression pattern of Meis2 in *Xenopus laevis* is of particular importance because of the clear progressive segmentation of the animal's brain, which allows a precise characterization of neuroanatomical boundaries and territories, and because it occupies an interesting evolutionary position. The expression pattern of Meis2 has been extensively characterized in amniotes, particularly mice, and zebrafish, but it is not known in detail in intermediate models such as anamniote tetrapods. Therefore, *Xenopus* represents an ideal evolutionary starting point to investigate how neuronal specification mechanisms are conserved or diversified in vertebrates. Detailed knowledge of the boundaries and divisions of the brain in *Xenopus* facilitates the correlation between gene expression and functional organization and helps identify both evolutionarily conserved functions and lineage-specific adaptations in the origin and diversification of neuronal types.

## 2 Materials and methods

### 2.1 Sequence analysis

Amino acid sequences for each taxon were obtained from the NCBI BLAST PROTEIN database using Meis2 orthologs provided by the NCBI Orthologs output for *Homo sapiens* (taxid: 9606) Meis2 (Uniprot: O14770), *Mus musculus* (taxid 10090) (NP_001153040.1), *Gallus gallus* (taxid 9031) (XP_046773660.1), *Trachemys scripta elegans* (taxid: 31138) (XP_034624833.1), *Xenopus laevis* (taxid: 8355) (XP_018087691.1), *Danio rerio* (taxid: 7955) (XP_009291615.1), and the cladistian fish *Polypterus senegalus* (taxid: 55291) (XP_039596916.1). The complete sequences of Meis1, Meis2, and Meis3 in *Homo sapiens* (NP_002389.1, O14770, and XP_024307383.1 accession numbers, respectively) and *Xenopus* (XP_018118301.1, XP_018087691, and NP_001081866.1 accession numbers, respectively) were compared, and these proteins were also compared with the sequence of the antigenic epitope. Sequence alignments were constructed using the alignment editor MegAlign Pro™ and the MUSCLE algorithm. Phylogenetic trees were generated and visualized using the BIONJ (Neighbor Joining) option and edited with Canvas X Draw. The branch length represented the average rate of change, measured in terms of changes per position.

### 2.2 Western blot

We performed western blotting analysis using the monoclonal mouse anti-Meis2 antibody (Santa Cruz Biotechnology Cat# sc-515470, RRID: AB_3076386) for *Xenopus laevis* and rat brain extracts. The anesthetized animals were perfused with cold saline (0.9% NaCl) to eliminate blood tissue. Then, the brains were removed and frozen in dry ice until use. The tissue was disaggregated in an equal volume of homogenization buffer [5 mM ethylenediaminetetraacetic acid (EDTA), 50 mM Tris pH 8, 150 mM NaCl, 10% glycerol, 1% Nonidet P40, 0.1% SDS; Roche, Mannheim, Germany] supplemented with protease inhibitors (SIGMAFAST™ Protease Inhibitor Tablets, # 8820). The protein content of the supernatants was calculated and diluted to apply 50 μg of protein in each lane. The samples of the rat (*Rattus norvegicus*; R.n) and *Xenopus laevis* (X.l.) brains, the purified human protein (3 μg of the human Meis2 recombinant protein with GST-tag at the N-terminal H00004212 P01.10UG. Abnova™), and the molecular weight (MW) standards (Prime Step Protein standard #773302 Biolegend) were run in a 12% polyacrylamide gel (#161-0801, Bio-Rad Laboratories, Inc., Hercules, CA, USA) and separated by SDS-PAGE electrophoresis using a Mini-Protean system (Bio-Rad). The separated samples were transferred to a nitrocellulose membrane and blocked for 30 min in WestVision Block and Diluent solution (SP-7000, Vector, Newark, CA, USA). The membrane was incubated at 4°C for 24h with the antibody diluted in Tris-buffered saline (TBS) containing 0.1% Tween-20 (antibody dilution 1:1,000; mouse anti-Meis2 antibody). After tree washes in TBS, the secondary goat anti-mouse antibody coupled to horseradish peroxidase (diluted 1:50,000; Jackson Immuno Research Laboratories, Inc., West Grove, PA, USA) was incubated for 2 hours at room temperature. After washing, it was revealed with ECL system (Western-Ready ECL Substrate Plus Kit ECL. #426316 Biolegend).

### 2.3 Animals and tissue preparation

The original research reported here was designed and carried out according to the ARRIVE guidelines and the regulations and laws established by the European Union (2010/63/EU) and Spain (Royal Decree 118/2021) for the care and handling of animals in research The experiments described herein were approved by the competent authority of the Complutense University [O.H. (CEA)-UCM-1615022024-2024] and the Community of Madrid (PROEX 087.0/24).

For the present study, embryos, larvae, and juvenile specimens of *Xenopus laevis* were used (see Supplementary Table 1). Developing specimens of *Xenopus laevis* were obtained by gonadotropin-induced breeding (SIGMA-MERK). The adult frogs were purchased from licensed suppliers (XenopusONE DX, Michigan, USA). *Xenopus laevis* embryos were staged according to Nieuwkoop and Faber (1967) as embryonic (30–45), premetamorphic (46–52), prometamorphic (53–58), and metamorphic climax larvae (59–65). At appropriate times, they were anesthetized by immersion in a 0.3% solution of tricaine methanesulfonate (MS222, Sigma-Aldrich Merck KGaA, Darmstadt, Germany; adjusted to pH 7.4) and fixed by immersion in the MEMFA [1 M 3-(N-morpholino) propanesulfonic acid (MOPS; Sigma-Aldrich), 20 mM EGTA (Sigma-Aldrich), 10 mM magnesium sulfate, and 4% formaldehyde, adjusted to pH 7.4.] fixative solution overnight at 4 °C. The adults, juveniles, and late larvae were perfused transcardially with 0.9% NaCl saline, followed by cold MEMFA fixative. The brains were cryoprotected in a solution of 30% sucrose in PB (0.1 M phosphate buffer, pH 7.4) for 4–6 h at 4 °C until they sank. They were then cut on a freezing microtome at 35 μm (adults) or 20–30 μm (juveniles and late larvae) in the transverse or sagittal planes, and the sections were collected in cold PB. The embryos were processed *in toto* for immunohistochemistry and finally sectioned at 14–16 μm thickness in the transverse, horizontal, or sagittal plane on a freezing microtome.

### 2.4 Immunohistochemistry

We performed an *in toto* procedure for the embryos up to stage 48, and free-floating sections were used from NF st49 to the adult specimens. Single immunodetection of Meis2 was conducted as follows: the first step of antigen retrieval was performed through an enzymatic procedure, where the sections or the embryonic brains were incubated in Proteinase K 20 μg/ml in TE Buffer, pH 8.0, for 15 min at 37 °C. Then, the samples were incubated in the blocking solution for 10 min at RT. After that, the samples were incubated for 48 h at 4°C with the primary mouse anti-Meis2 antibody (RRID: AB_3076386) diluted 1:500 in PBT (PB 0.5% Triton X-100), followed by a second incubation for 90 min at room temperature with the DyLight 488-conjugated horse anti-mouse antibody (Vector Laboratories, DI-2488) diluted 1:500 in PBT.

In addition, we performed double immunolabeling experiments by combining, in the first incubation, the anti-Meis2 antibody with other antibodies (see Table 1 for specifications) against tyrosine hydroxylase (TH), calcium-binding protein calbindin (CB), calretinin (CR), orthopedia (Otp), Satb2, and Pax6. For the second incubation, the combination of the DyLight 488-conjugated horse anti-mouse antibody (Vector Laboratories, DI-2488) and the DyLight 594-conjugated goat anti-rabbit antibody (Vector Laboratories, DI-1594) or the Alexa 594-conjugated donkey anti-goat antibody (1:300, Invitrogen, Thermo Fisher Scientific, A11058) was used.

**Table 1:** Primary antibodies used in the study.

**Name**	**Immunogen**	**Commercial supplier**	**Dilution**
Meis2	Amino acids 45–63 near the N-terminus of human Meis2. Clone ID H-10	Santa Cruz Biotechnology Cat# sc-515470, RRID:AB_3076386	1:500
Satb1	Amino acids 241–310 of human SATB1	Santa Cruz Biotechnology Cat# sc-376096, RRID:AB_10986003	1:500
Pax6	Peptide sequence: QVPGSEPDMSQYWPRLQ of the C-terminus of the mouse PAX6 protein Clone: *P*ol*y1*9013	Polyclonal rabbit anti-Pax6; Biolegend (previously Covance). Cat# 901301 AB_2565003	1:300
Otp	Amino acid sequence: RKALEHTVS of the C-terminal OTP protein	Polyclonal rabbit anti-Otp. Pikcell Laboratories, Kruislaan, Amsterdam, The Netherlands. RRID:AB_2315023	1:500
CB	*Escherichia coli*-produced recombinant rat calbindin D-28k	Polyclonal rabbit anti-calbindin D-28; (Swant Cat# cb38a, RRID:AB_3107026)	1:500
CR	*E. coli*-produced recombinant human calretinin	Polyclonal rabbit anti-calretinin; (Swant Cat# 7699/4, RRID:AB_2313763)	1:1,000
TH	Protein purified from rat pheochromocytoma	Polyclonal rabbit anti-TH; Merck- (Millipore Cat# AB152, RRID:AB_390204)	1:1,000

After the immunohistochemical procedures, the embryos were sectioned as described above and directly mounted onto glass slides. The free-floating sections were rinsed in PB and then mounted. All slides were then coverslipped with a fluorescence mounting medium containing 1.5-μg/ml 4′,6-diamidino-2-phenylindole for DNA counterstaining (UltraCruz, SC-24941, Santa Cruz, Dallas, TX, USA).

### 2.5 Controls and specificity of the antibodies

**Figure 1:**
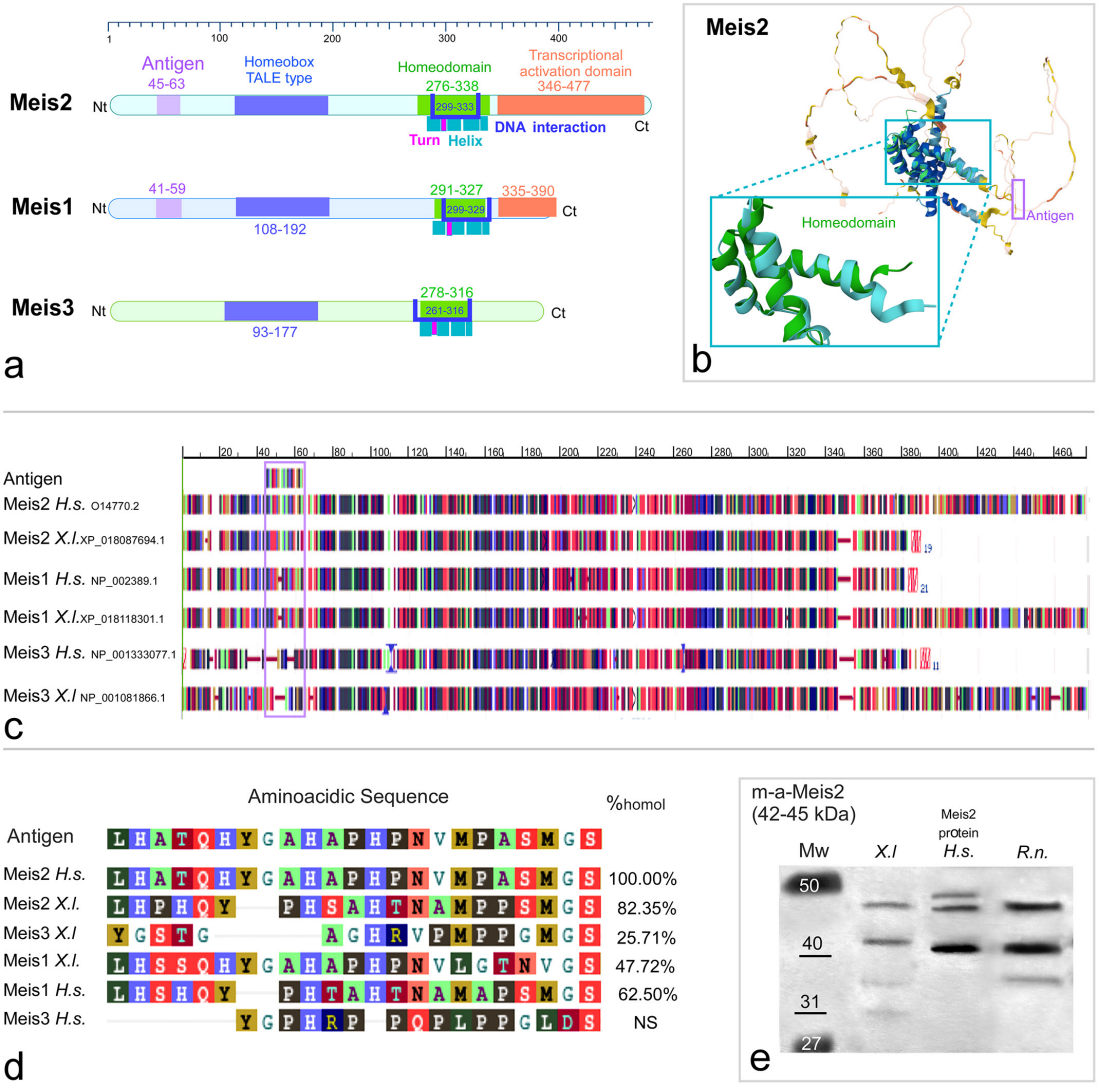
**(a)** Representation of the structure and main domains of the human Meis2, Meis1, and Meis3 proteins. The domains are labeled in Meis2 and the color code is maintained in Meis1 and Meis3. **(b)** The 3D structure obtained from the AlphaFold Protein Structure Database and PDBe-KB (Protein DATA Bank in Europe Knowledge Base) for human Meis2 Uniprot Id: O14770.2 Entrez Gene ID: 4212. The central part, including the homeodomain (cyan rectangle) and the Nt loop, contains the antigenic sequence recognized by the antibody used (violet rectangle). **(c)** Alignment of the Meis1, Meis2, and Meis3 orthologs between *Homo sapiens* and *Xenopus laevis*. **(d)** Percentage of homology with the amino acid sequence of the antigenic epitope for the Meis1, Meis2, and Meis3 orthologs in *Xenopus laevis* and *Homo sapiens*. **(e)** Western blot analyses of brain extracts from *Xenopus* (*X.l*), dilution of the purified Meis2 human protein (*H.s*), and *Rattus norvegicus* (*R.n*), stained with the mouse anti Meis2 antibody. Standard molecular weight (MW) is represented on the left side of the photograph.

The suitability and specificity of the Meis2 antibody used in the present study, generated against the protein corresponding to the human Meis2 Gene ID: 4212 (Uniprot: O14770), were assessed by the commercial supplier. Its specificity was also demonstrated *in silico* and tested using western blot analysis (see sequence analysis and western blot sections in Materials and methods and Results). The predicted MW of the target protein (NM_172316) in the western immunoblotting (WB) experiment is 33.46–40 kDa in *Homo sapiens* (as seen in the specification sheet of the provider). The predicted molecular weight of Meis2 is between 42 and 43kDa in rats and between 42 and 44.73 kDa in *Xenopus*, depending on the isoform, in a denatured SDS-PAGE gel. The western blot analysis showed a lane in the predicted molecular weight in both the rat and *Xenopus* lanes (Figure 1e).

Before proceeding with the staining protocol, we performed a blocking control by neutralizing the antibody with 5 μg of the human Meis2 full-length ORF recombinant protein with a GST-tag at the N-terminal (H00004212 P01.10UG. Abnova™) diluted in PB for each 1 μg of the antibody. The control and blocking tubes were incubated for 20 min at room temperature and used in the first step of immunohistochemistry. In all cases, a total loss of labeling was observed in the tissue.

All other antibodies have been extensively characterized in this species in previous studies (Morona and González, 2008; Dominguez et al., 2013; Bandin et al., 2013, 2014; Moreno et al., 2014; Morona et al., 2011, 2017). Prior to all incubations with the second antibody cocktails, the sections were incubated for 1 h at 24 °C in normal sera of the species from which the secondary antibodies were obtained.

Finally, all control experiments involved parallel incubation of alternate sections either with the antiserum or with the omission of the primary or secondary antiserum. No labeling was detected.

### 2.6 Evaluation and presentation of the results

**Figure 2:**
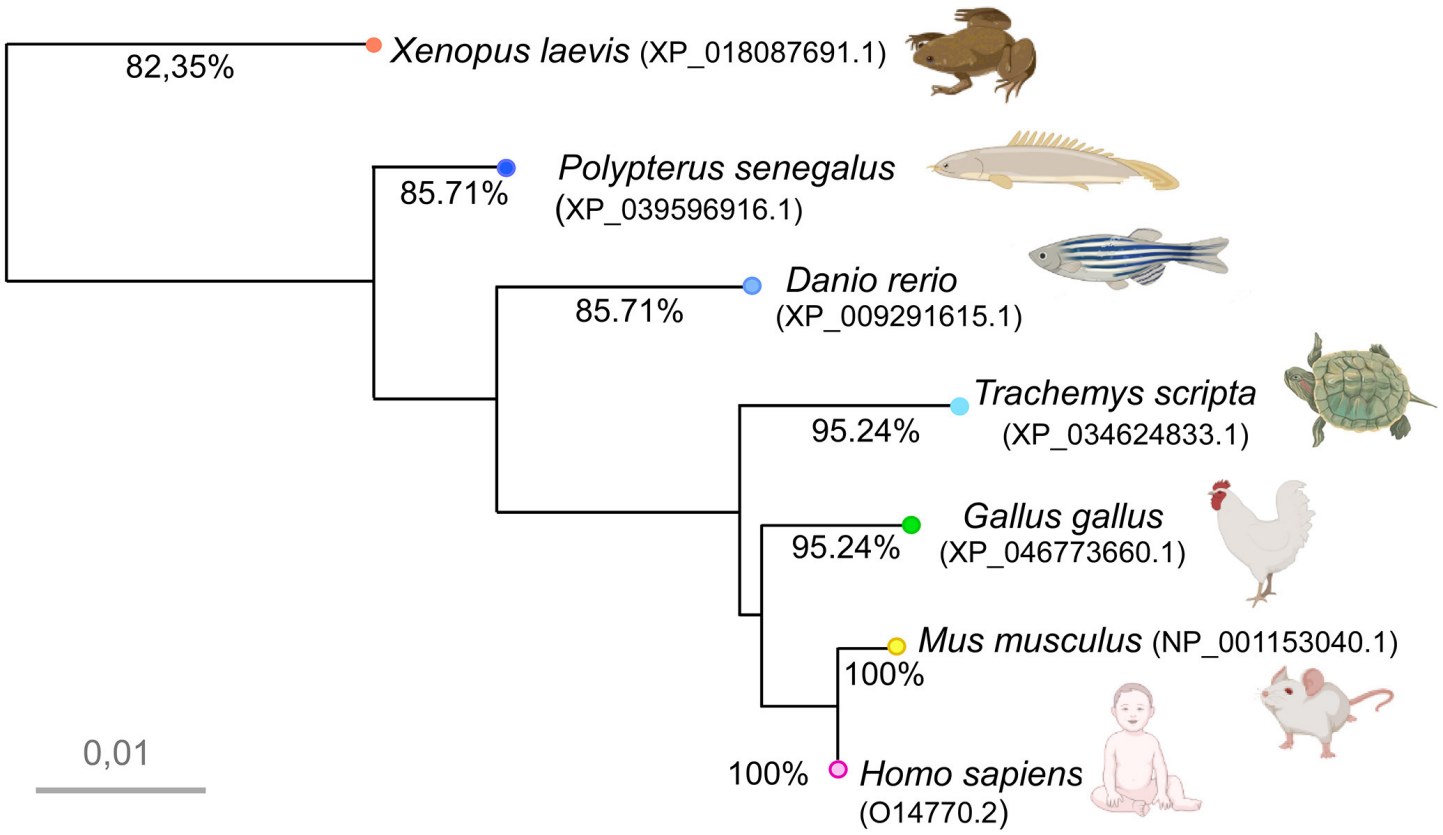
Phylogenetic tree illustrating the relationships among the amino acid sequences of the transcription factor Meis2 in the indicated animal models based on a COBALT multiple alignment of the human Meis2 orthologs, as defined by the NCBI orthologs criteria. The accession number of each ortholog is provided under the species name in the diagram. The scale bar represents distance, and the percentage of calculated homology with human Meis2 (Uniprot Id: O14770.2) is shown in each terminal branch. Free images were obtained from BioRender or Adobe Stock and modified using CANVAS.

**Figure 3:**
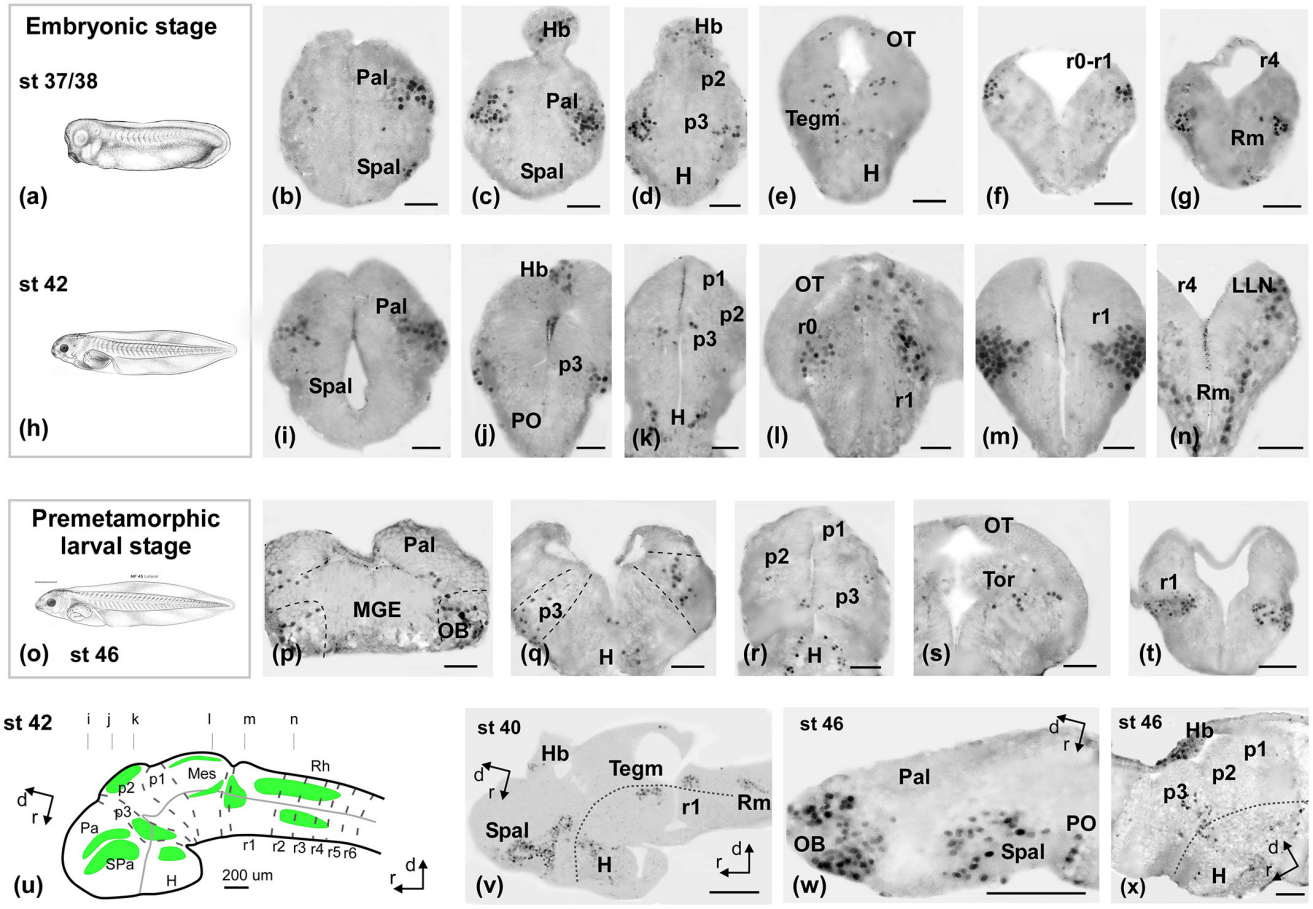
Photomicrographs of Meis2-ir transverse **(a–t)** and sagittal **(u–x)** sections at representative brain levels of *Xenopus* during embryonic stages. Topological dorsal and rostral orientations are indicated by rows, with respect to the alar-basal boundary (ABB). Stages 37–38 **(a–g)**, 42 **(h–n)**, and 46 **(o–q)** are indicated on each photomicrograph. The approximate levels of transverse sections are indicated in **(u)**. Dashed lines mark the approximate boundary between neuromeres and telencephalic subdivisions. See the list for abbreviations. Scale bars = a–n =50 μm, p–t, v–x = 100 μm = 200 μm.

**Figure 4:**
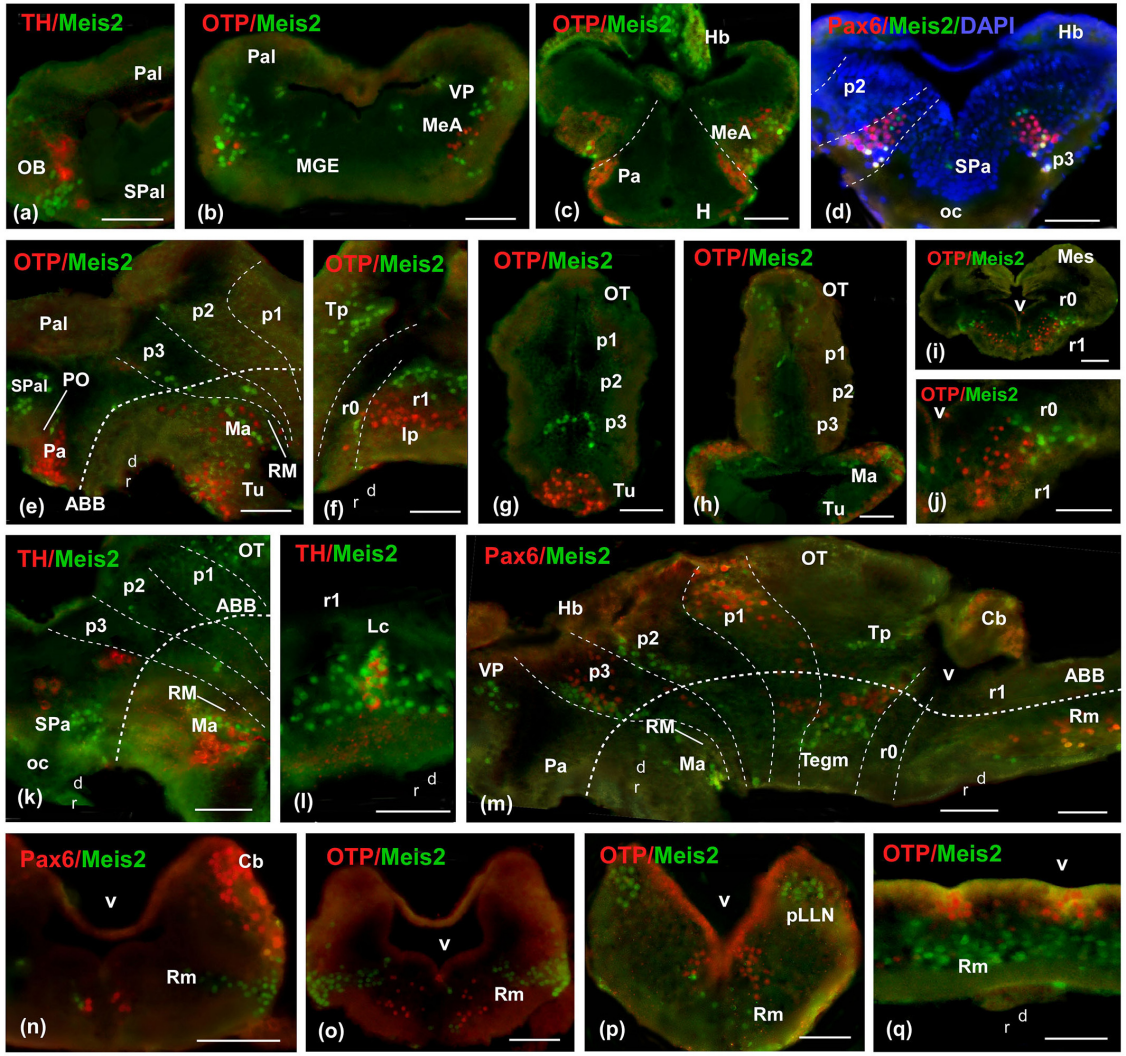
Photomicrographs of double-labeled transverse **(a–d, g–j, n–p)** and sagittal **(e, f, k–m, q)** sections at representative brain levels of *Xenopus* from stages 42 to 50. Topological dorsal and rostral orientations are indicated by rows, with respect to the alar-basal boundary (ABB), indicated by a rough dashed line in sagittal sections. Color codes for the markers are indicated in the upper left corner of each photo. Thin dashed lines represent the approximate boundaries within telencephalic, diencephalic, mesencephalic, and rhombencephalic subdivisions. See the list for abbreviations. Scale bar p–x = 100 μm, u = 200 μm, others = 50 μm.

**Figure 5:**
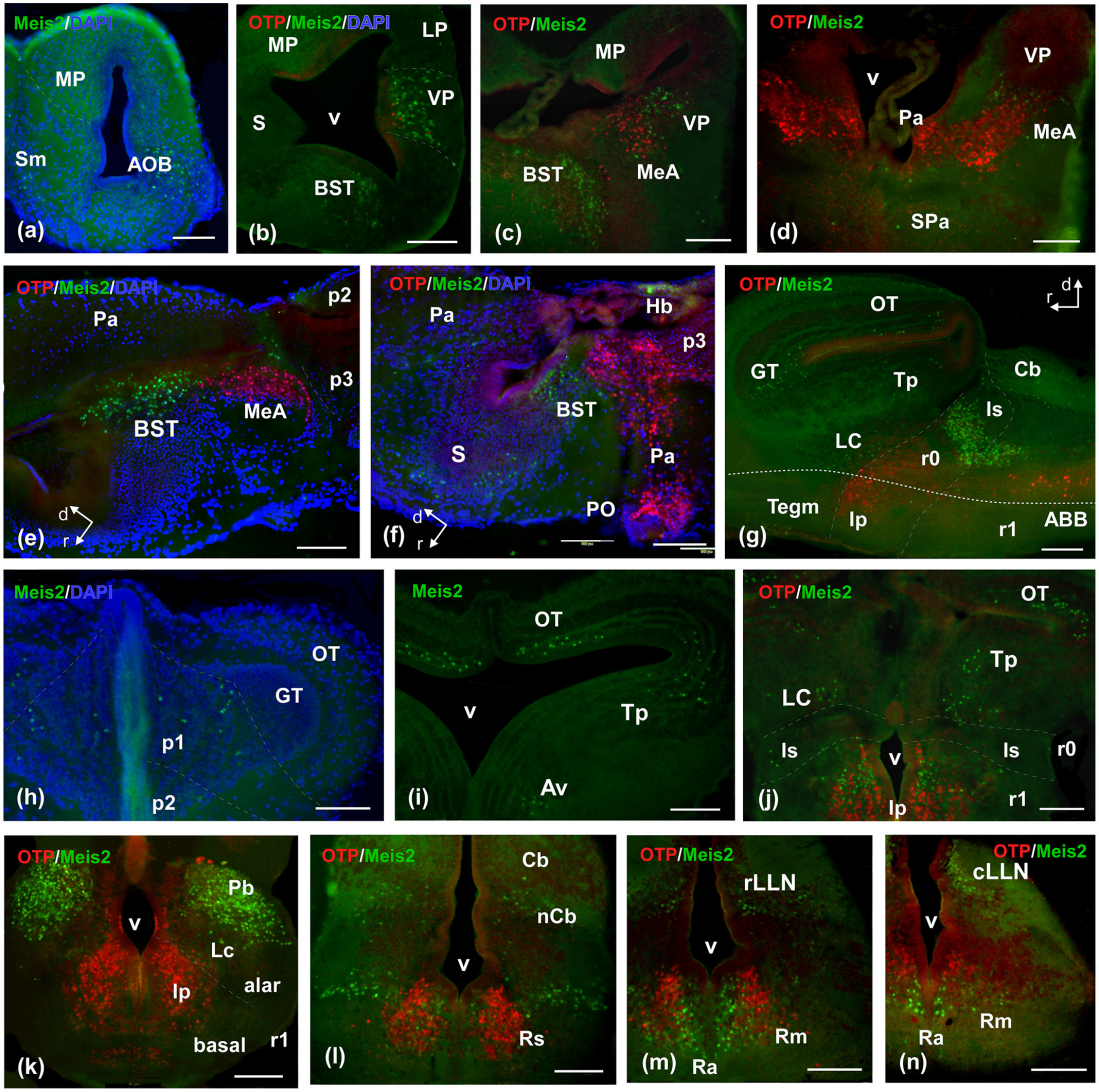
Photomicrographs of double-labeled transverse **(a–d, h–n)** and sagittal **(e–g)** sections at representative brain levels of *Xenopus* from stages 53–56. Topological dorsal and rostral orientations are indicated by rows, with respect to the alar-basal boundary (ABB), indicated by a rough dashed line in sagittal sections. In all photographs, green is Meis2ir, red is Otp, and blue is DAPI staining. Thin dashed lines represent the approximate boundaries within telencephalic, diencephalic, mesencephalic, and rhombencephalic subdivisions. See the list for abbreviations. Scale bar, all = 100 μm.

**Figure 6:**
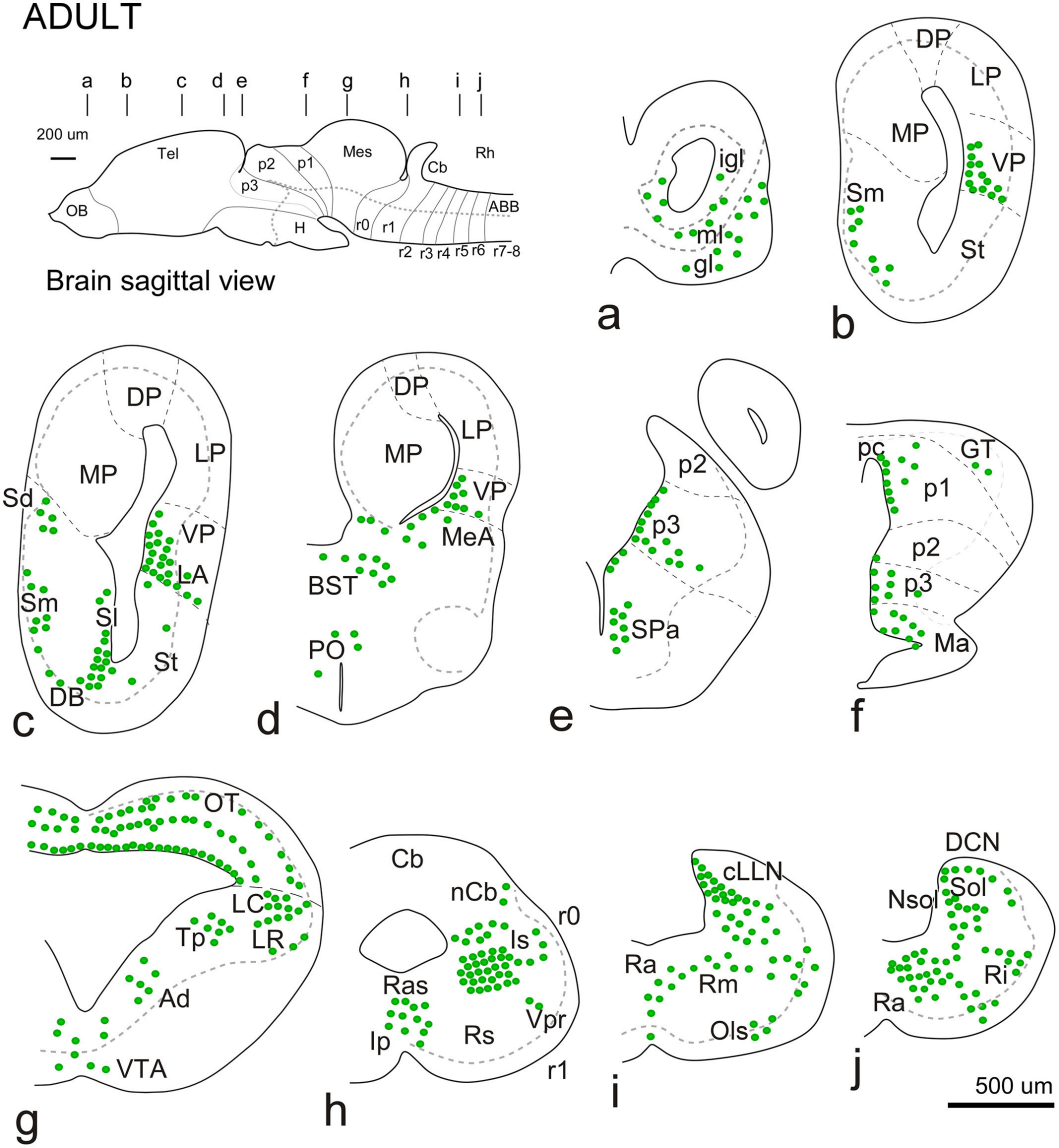
Schematic diagrams showing the rostrocaudal distribution of Meis-2ir in the adult brain. In all diagrams, dorsal is oriented upward. The main subdivisions are indicated by dashed lines. The approximate levels are indicated in the upper sagittal scheme. See the list for abbreviations.

**Figure 7:**
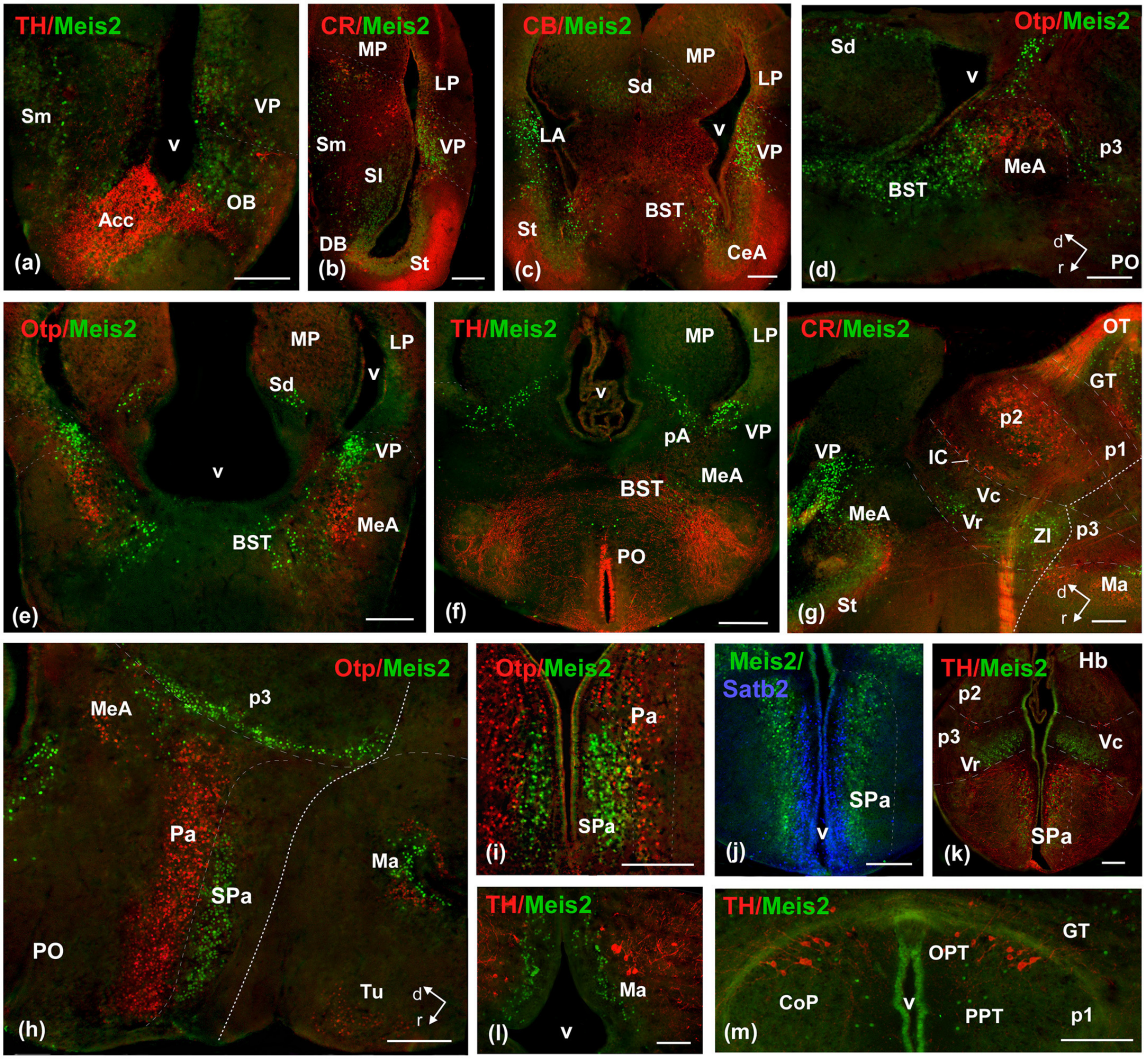
Photomicrographs of double-labeled transverse **(a–c, e, f, i–m)** and sagittal **(d, g, h)** sections at representative brain levels of *Xenopus* from stages 53–56. Topological dorsal and rostral orientations are indicated by rows, with respect to the alar-basal boundary (ABB), indicated by a rough dashed line in sagittal sections. Color codes for the markers are indicated in the upper left corner of each photo. Thin dashed lines represent the approximate boundaries within telencephalic, diencephalic, mesencephalic, and rhombencephalic subdivisions. See the list for abbreviations. Scale bar, all = 100 μm.

**Figure 8:**
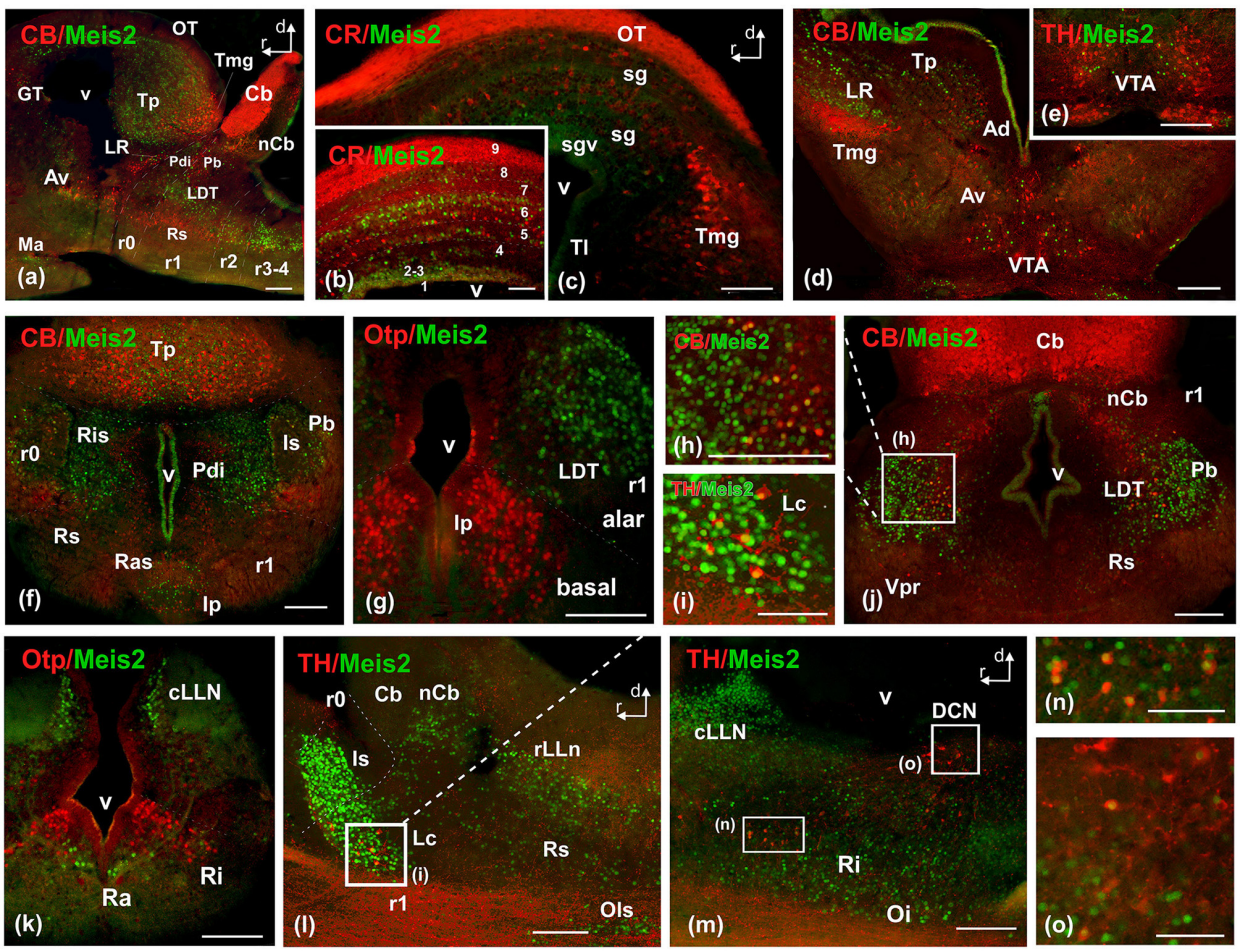
Photomicrographs of double-labeled transverse **(a–c, e, f, i–m)** and sagittal **(d, g, h)** sections at representative brain levels of Xenopus from stages 53–56. Image **(h)** is a magnification of **(j)** at the level of the laterodorsal nucleus. **(i)** is a magnification of the locus ceruleus in **(l)**. Images **(n)** and **(o)** are magnifications of **(m)** at the reticular formation and DCN respectively. Topological dorsal and rostral orientations are indicated by rows. The alar-basal boundary (ABB) is indicated by a rough dashed line in sagittal sections. Color codes for the markers are indicated in the upper left corner of each photo. Thin dashed lines represent the boundaries within telencephalic, diencephalic, mesencephalic, and rhombencephalic subdivisions. See the list for abbreviations. Scale bar, no = 25 μm, rest = 100 μm.

**Figure 9:**
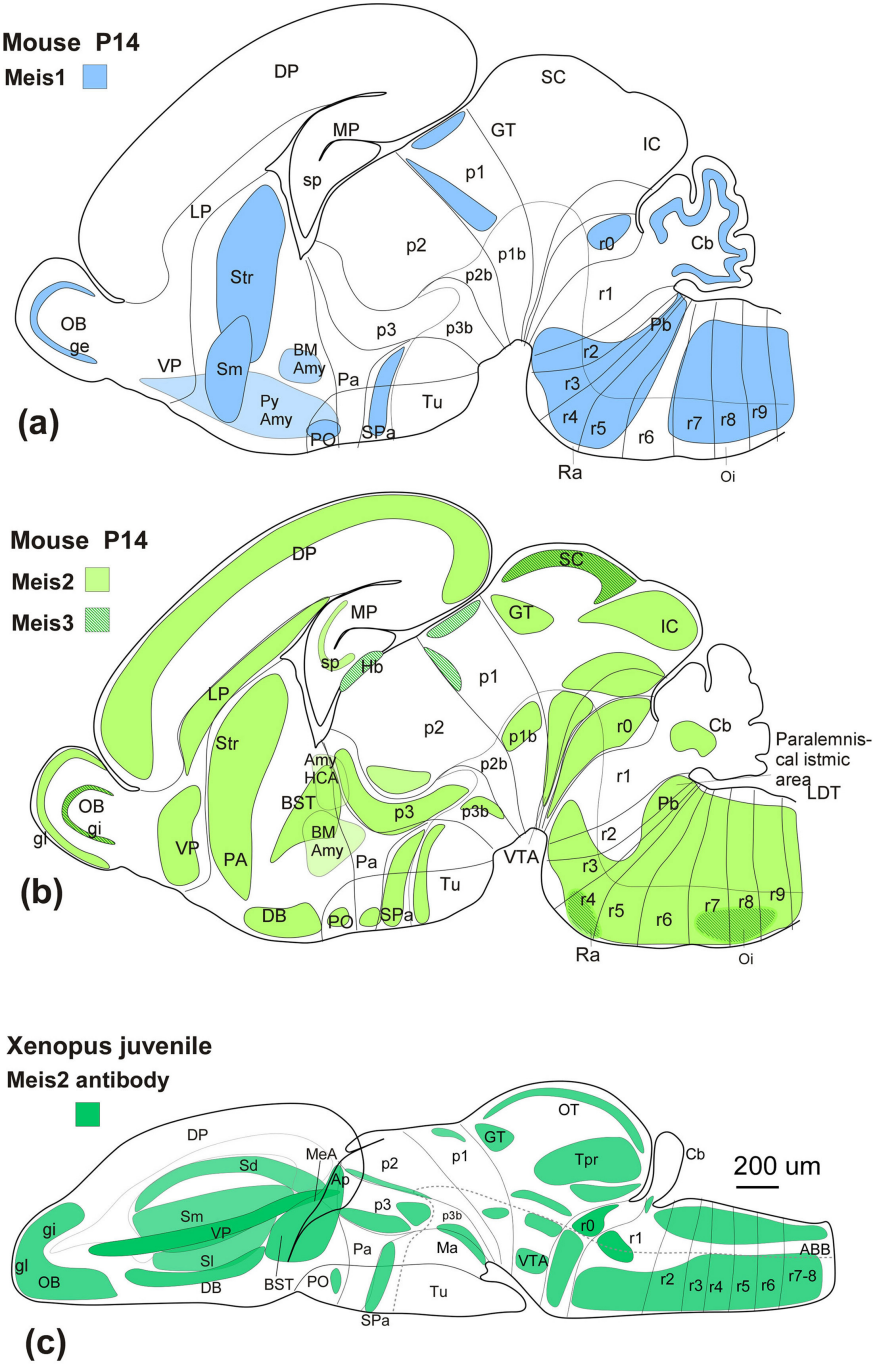
**(a)** Sagittal schematic diagrams representing the distribution of Meis1 (blue), **(b)** Meis2 (green), and Meis3 (dark green lines) in the mouse postnatal brain (Allen brain), compared to the distribution of Meis2-ir in the *Xenopus* brain **(c)**. In all diagrams, dorsal is oriented upward and rostral is oriented to the left. The main subdivisions are indicated by thin lines. See the list for abbreviations.

**Figure 10:**
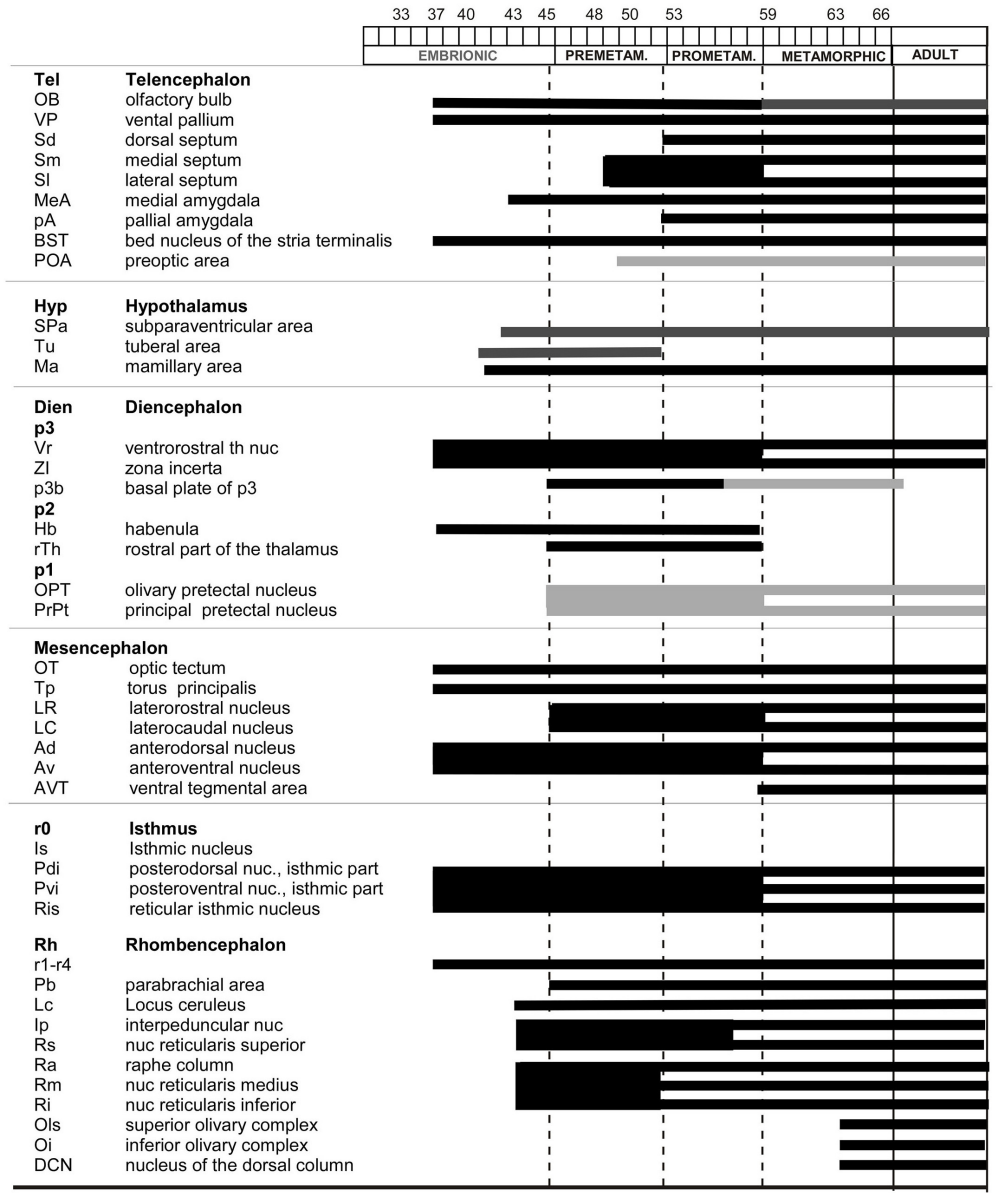
Timeline of the appearance of Meis2 cell groups in the developing brain of *Xenopus laevis*.

The localization of the Meis2-ir populations detected in the present study was framed within the neuromeric model (Puelles and Rubenstein, 2003, 2015), adapted for the brain of *Xenopus laevis* for each developmental period (Moreno et al., 2008a,b,c,d; Morona and Gonzalez, 2013; Bandin et al., 2013, 2015), and the nomenclature used was the same as that in previous studies. The sections were examined under an Olympus BX51 microscope (Olympus, Hamburg, Germany). Representative photomicrographs were captured with a digital camera (Olympus DP74), processed using Adobe Photoshop (Adobe Systems, San Jose, CA, USA), and the final figure plates were assembled in Canvas X Draw (Canvas GFX). To facilitate understanding of the distribution of Meis2-immunoreactive (Meis2-ir) cells at each developmental stage, their locations were charted in sagittal and transverse schematics covering rostral to caudal brain levels, also created in Canvas X Draw. Protein analysis was first conducted (Figures 1, 2), followed by the presentation of results arranged sequentially from embryonic to larval premetamorphic stages (Figures 3, 4), prometamorphic stages (Figure 5), and metamorphic and adult stages (Figures 6–8). The comparison of Meis2 expression between Xenopus and mouse is shown in Figure 9. Finally, the temporal sequence of appearance is summarized in Figure 10.

## 3 Results

### 3.1 Protein analysis

The antibody used in this study was raised in a mouse against amino acids 45 to 63 of human Meis2 (477aa). Meis2, together with Meis1 and Meis3, is a member of the TALE superclass of homeodomain proteins that share an atypical homeodomain known as “TALE” and the MEINOX domain characteristic of Meis proteins (Bürglin, 1998). The DNA-binding homeodomain is approximately 60 amino acids long and consists of three alpha helices (Gehring et al., 1994). The third alpha helix is the primary DNA-binding region, although there are other DNA contacts outside helix three (Passner et al., 1999). Moreover, they have an additional transactivation domain (amino acids 346–477), which is shorter in Meis1 and absent in Meis3 (Figures 1a, b). These members share similar domains; therefore, we compared them to evaluate the degree of homology and the potential antibody specificity in species other than humans. The comparison of the epitope sequence of the human Meis2 protein with those of the *Xenopus* Meis2, Meis1, and Meis3 proteins (Figure 1c) revealed homology percentages of 82.35%, 47.42%, and 25.71%, respectively (Figure 1d). In addition, the comparison of the epitope with the human proteins showed 100% coincidence for Meis2, 62.50% for Meis1, and no coincidence for Meis3. Therefore, in *Homo sapiens*, cross-reactivity of the antibody with Meis1 is not possible, and it would be very unlikely in *Xenopus*.

Western blot with the mouse anti-Meis2 antibody against the brain extract of *Xenopus laevis (X.l)*, and *Rattus norvegicus (R.n)*, and compared with the purified Human Meis2 recombinant protein (H00004212 P01.10UG. Abnova™; H.s) showed several bands in each lane. In the lane of purified human protein three bands were observed at approximately 42, 45, and 48 KDa were stained for the Xenopus and rat brain extracts. The expected molecular weight for *Xenopus* Meis2 was 42–50, depending on the isoform.

The phylogenetic tree based on this COBALT alignment of human, mouse, chicken, *Pseudemys, Xenopus, Zebrafish*, and *Polypterus* orthologs reveals high homology within the conserved domains with respect to the ancestral state. This analysis also revealed a high degree of identity among the orthologs in amniotes, over 95% in all cases, with minimal variation (branch length less than 0.01), whereas higher variation was observed between amniotes and anamniotes (Figure 2). In particular, the MEINOX and homeodomain of *Xenopus* and the human Meis2 protein were 97.5 and 98.4% identical, respectively. Notably, *Xenopus* appeared as a basal state, while *Polypterus* formed a separate branch within the actinopterygians due to dissimilarities in the sequence.

### 3.2 Meis2 immunofluorescence brain distribution

The staining pattern described below refers to the Meis2 immunoreactivity (Meis2-ir) observed in the central nervous system of *Xenopus laevis*. The Meis2 expression pattern in the developing brain was studied from the early embryonic stages (st) to the metamorphic climax, and the labeling pattern was consistent throughout the developmental sequence and among the animals. For the description of the results, development is subdivided into embryonic (st 32–42), premetamorphic (st 46–52), prometamorphic (st 53–59), and climax-juvenile (st 60 to 1 year) periods (see Supplementary Figure 1). The appearance of progressive or transient immunoreactive cell groups in the developing brain of *X. laevis* is summarized in Figure 10.

#### 3.2.1 Embryonic and premetamorphic stages

The immunoreactive (-ir) cells detected at the embryonic stages (Figure 3) in the telencephalon were located in two continuous bands along the ventrolateral pallium and the subpallium (Figures 3b, c, i, v). In the hypothalamus, some cells were sparsely distributed in the alar and basal portions (Figures 3k, v). In the diencephalon, scattered cells were observed in prosomere 3 (p3; Figures 3d, j, k, v) and the habenula (Figures 3c, d, j). A group of faint cells was observed in the optic tectum (OT; Figures 3e, l, v) and in the mesencephalic tegmentum (Tegm; Figures 3e, v). In the rhombencephalon, the most conspicuous group appeared, which occupied the lateral rim around the isthmic region (Figures 3f, g, l, m, v) and extended along the alar and basal plates in rhombomeres r1–r4 (Figures 3g, n, v; Supplementary Figures 2, 3).

From the premetamorphic stages onward, the olfactory bulb (OB) showed a conspicuous population of Meis2-ir, distinct from the TH-ir cells observed through double immunofluorescence (Figures 3p, w, 4a). The pallial population was identified in the ventral part of the pallium (Figures 3p, 4b). In the subpallium, an intense band was detected in the medial ganglionic eminence (MGE; Figures 3p, w, 4b), and the prospective medial amygdala (MeA), identified by the Otp-ir, depicted scattered Meis2-ir (Figures 4b, c). Finally, in the preoptic area (PO), scattered Meis-ir cells were detected (Figures 3w, 4e). In the hypothalamus, Meis2-ir cells were observed in the alar (Figures 3q, w) and basal portions (Figures 3r, x). In the alar hypothalamus, these cells occupied the subparaventricular region, as indicated by colocalization with Pax6-ir (Figure 4d) and TH-ir (Figure 4k). In the basal hypothalamus, they were found in the prospective tubero-mamillary area, identified by co-labeling with Otp (Figures 4e, g), TH-ir (Figure 4k), and Pax6-ir (Figure 4m).

In the diencephalon, Meis2-ir was present in a band of cells in p3, as revealed by the double immunohistochemistry with Pax6-ir in the alar plate, where punctual double labeled Meis2/Pax6-ir cells were detected (Figures 4d, m). In the basal plate, Meis2 cells were labeled in continuity with the mammillary region (Figures 4e, m). In prosomere 2 (p2; Figures 3r, 4e, m), including the rostral part of the habenula (Figures 3x, 4c), Meis2-ir cells were observed. At the mesencephalic level, Meis2-ir populations were distinguished in the OT and the toral and tegmental regions (Figures 3s, 4f, m). In the rhombencephalon, the conspicuous population in r0 and r1 progressively invaded medial areas (Figures 3t, 4i, j). The TH-ir co-labeling demonstrated the presence of Meis2-ir cells in the locus coeruleus (Lc; Figure 4l). Finally, Meis2-ir cells spread along the alar and basal plates from r2 (Figures 3t, 4n–q), within the medial areas of the raphe, in the reticular formation, and in the developing octavolateral area (Supplementary Figure 4).

#### 3.2.2 Prometamorphic larvae

The prometamorphic larval stages are marked by important changes, including the development of the hind limbs and other adaptations that are reflected in the nervous system. By this period, the brain shows most of its main subregions and a segregation of most cell groups according to the adult configuration. In the OB, Meis2-ir cells were observed in the main and accessory structures (Figure 5a). In the pallium, Meis2-ir was restricted to the ventral pallium (Figures 5b, c), and caudally, scattered cells were adjacently observed, by co-labeling with Otp, in the MeA (Figures 5c–e). In the subpallium, abundant Meis2-ir cells were observed in the bed nucleus of the stria terminalis (BST; Figures 5b, c, e, f) and scattered in septal territories (Figures 5a, f).

The basal hypothalamus exhibited a similar pattern to that observed in previous stages. The diencephalic population in p2 was less intense, and it was still present in the habenula (Figure 5f). Both p3 and the commissural subdivision of p1 exhibited a higher number of cells (Supplementary Figure 5; Figure 5h). The griseum tectale (GT) showed a Meis2-ir population, whereas the tectal population was grouped in the medial part of the OT, without a clear layered pattern (Figures 5g, i). Caudally, toral and tegmental populations were evident (Figures 5g, i, j). During this period, rhombencephalic populations were prominent and expanding. The intense group detected in r0 and r1 extended caudally and medially in the direction of the reticular isthmic nucleus, the superior reticular formation, and the scattered cells in the interpeduncular nucleus (Ip; Figures 5g, j, k). In r1, some cells were found in the cerebellar nucleus (nCb) and in the area of the principal sensitive trigeminal nucleus (Figure 5l). Caudally, Meis2-ir cells extended along the entire alar plate. They were particularly prominent along the rostral and caudal lateral line nuclei (LLN) and in the basal plate, along the medial band, in the raphe column, and longitudinally within the reticular nuclei (Figures 5m, n; Supplementary Figure 5).

#### 3.2.3 Metamorphic climax and adult

During the metamorphic climax, the tadpole becomes a juvenile, characterized by the resorption of the tail and a shift in locomotion to limb-based movement. In this rapid period (around 10–12 days), the froglet attains an adult-like brain configuration and shows a similar pattern of Meis2-ir staining; therefore, both stages will be described in the same section.

The general distribution pattern was maintained compared to the previous stages, except for the absence of Meis2-ir in p2 and the habenula and a broader extension along the rhombencephalic populations. In addition, at these stages, Meis2-ir populations were identified more precisely within the adult nuclear configuration (Figure 10). In the OB, Meis2-ir cells were observed (Figures 6a, 7a). Along the telencephalon, Meis2-ir cells were present in the ventropallial region (VP; Figures 6b–d, 8a–e). Scattered cells were observed caudally at the level of the anterior commissure in the MeA (Figures 7d, e) and, more conspicuously, in the pallial amygdala (pA; Figure 7f). In the subpallium, Meis2-ir cells were observed scattered from the diagonal band, more intense in the BST, and only occasionally detected in the striatum (Figures 6c, d, 7b, c–e). However, in the septum, along the rostrocaudal extent of the telencephalon, scattered Meis2-ir cells were observed in the medial, lateral, and dorsal portions (Figures 6b, c, 7a–e). Scarce cells were found in the preoptic area (Figures 6d, 7f). In the alar hypothalamus, double labeling with Otp, Satb2, and TH identified Meis-ir cells in the SPa (Figures 6e, 7h–k), whereas in the basal hypothalamus, Meis2-ir cells were observed in the mamillary region (Figures 6f, 7l). The group localized in p3 extended to the ventro-rostral prethalamic nucleus (Vr) and the nucleus of the zona incerta (ZI; Figures 6e, f, 7g, h, k). No Meis2-ir cells were found in p2 during this period. In p1, some cells were present in the commissural domain (CoP), corresponding to the olivary pretectal nucleus (OPT), TH-ir +, and part of the principal pretectal nucleus (PrPt, Figures 6f, 7m). Faint cells were also labeled in the basal plate of this prosomere.

In the mesencephalon, Meis2-ir cells were observed in the GT (Figures 6f, 7m, 8a). In the OT, Meis2-ir cells were mainly found in layers 1–3 and 6 and scattered in layers 4, 5, and 8 (Figures 6g, 8a, b). Meis2-ir cells in the torus principalis exhibited strong immunoreactivity, and the populations in the laterorostral (LR) and laterocaudal (LC) nuclei were more abundant than at earlier stages (Figures 6g, 8a, d). Additional Meis2-ir cells were found in the anterodorsal (Ad) and anteroventral (Av) tegmental nuclei, and in the midline, Meis2-ir cells were found in the ventral tegmental area (VTA) that did not colocalize with TH (Figures 6g, 8d, e).

The groups found in r0 and r1 constituted the most intense and numerous Meis2-ir populations in the brain. During this period, in r0, it extended to surround the isthmic nucleus along the peri-isthmic areas, including the isthmic reticular formation (Ris), the parabrachial area (Pb), the posterodorsal isthmic nucleus (Pdi), and, basally, the interpeduncular nucleus (Figures 6h, 8f–j). This population continued into r1 and included the Lc, which co-expressed TH, and was scattered throughout the laterodorsal tegmental nucleus (LDT; Figures 6i, 8g–j, l). Additional rhombencephalic populations exhibited a pattern similar to that of the previous period, including the cerebellar nucleus and the reticular formation (Figures 8f, j, l, m). Meis2-ir extended along the rest of the hindbrain, the rostral and caudal nuclei of the lateral line, the raphe nuclei, and the superior olivary complex (Figures 6h–j, 8k–m). Notably, TH-ir populations in the reticular formation and dorsal column nucleus (DCN) were double-labeled for Meis2-ir (Figures 8m, o; see Supplementary Figure 6).

## 4 Discussion

### 4.1 Evolutionary conservation of the Meis family members

Members of the Meis family in vertebrates, including Meis1, Meis2, and Meis3, play essential roles in central nervous system regionalization and neuronal differentiation in vertebrates through a shared DNA-binding domain that determines their interactions (Ferretti et al., 2000). From an evolutionary perspective, the three Meis genes show a high degree of conservation, but in particular, the analysis of the antigenic sequence of the Meis2 antibody used in the present study reveals that Meis2 and Meis1 share a larger proportion of this sequence, while Meis3 shows minimal similarity. In this regard, our western blot analyses and the antibody blocking experiments with the full-length human Meis2 protein (resulting in a total absence of labeling) confirmed the specificity of the antibody in detecting Meis2 in *Xenopus laevis*, which experimentally supports our expression results. Given the differences in the sequence homology of the antigenic region of Meis2 (87.35%) and Meis1 (47.72%), cross-detection of the antibody is not expected, although it cannot be completely ruled out (see Figure 1). However, the expression pattern observed in *Xenopus laevis*, compared to that observed in mice (see Figure 9), further supports that the immunoreactivity observed corresponds to that of Meis2.

### 4.2 Meis members in regionalization and neuronal differentiation of the central nervous system

According to data from the Allen Brain Atlas of mouse development, Meis1, Meis2, and Meis3 are differentially expressed throughout the nervous system from early stages. Meis2 shows early expression from E11.3 in pallial and subpallial regions, with prominent signals observed in the alar plate of p3, the basal plate of p1, the optic tectum, and the mesencephalic tegmentum. In the hindbrain, its expression is detectable in the deep nuclei of the cerebellum and in the basal plate of r2. At E18.5, Meis2 is found in the olfactory bulb and all subdivisions of the pallium. It is particularly abundant in the striatum (Yang et al., 2021), medial septum, preoptic area, suprachiasmatic region, and the alar region of p3. It is also highly expressed in the midbrain, particularly in the inferior colliculus and tegmentum, and in the basal plate of r2. Comparatively, Meis1, at E11.3, is expressed in the subpallium, the hypothalamic peduncular paraventricular area, the alar plate of p3, the rostral precommissural domain of p1, and the alar and basal plates of the hindbrain. Later, between E13 and E15, Meis1 is also detected in the olfactory bulb, the medial and lateral ganglionic eminences, alar hypothalamic regions, and the alar plate of p3. Scattered cells are also observed in the optic tubercle and preisthmic and isthmic nuclei, with a higher expression level in the hindbrain. This expression decreases in density from E15 to the postnatal period, but it expands in distribution, where it is detected in the piriform and entorhinal cortex, as well as in the basomedial amygdala, striatum, and the granular layer of the cerebellum. In contrast, Meis3 is weakly expressed in the midbrain and the hindbrain basal plate at E11.5 and E13.5. It has also been observed in the hippocampus and the granular layer of the cerebellum, although its expression profile is less robust compared to Meis1 and Meis2 (Allen Brain Atlas).

Therefore, when comparing the expression of the three members of the Meis family, Meis1 and Meis2 show a very similar pattern and differ significantly from Meis3, especially at more advanced stages (see Figure 10). However, Meis1 and Meis2 show notable differences, such as the exclusive Meis1 expression in the granular layer of the cerebellum, where it appears to play an important role in the molecular processes of neurogenesis, as it upregulates cerebellar proneural factors such as Pax6 and BMP signaling and promotes the activation of SMADS and the degradation of Atoh1 (Owa et al., 2018). The absence of Meis2-ir in this region in *Xenopus* (see Figure 9) further reinforces that the observed pattern corresponds exclusively to Meis2.

The high degree of conservation of Meis2 among vertebrates, as evidenced by strong sequence homology with orthologs from mice, chickens, turtles, and various actinopterygian fish, suggests that its roles in the central nervous system are evolutionarily conserved, despite certain differences in species-specific expression patterns (which we will discuss below). The spatiotemporal pattern of Meis2 development described in zebrafish is consistent with that observed in *Xenopus*, further supporting the results presented. These findings stress the importance of Meis2 in regional specification and neuronal differentiation along the neuraxis (Biemar et al., 2001; Zerucha and Prince, 2001).

### 4.3 Expression patterns and anatomic regionalization

In the mouse olfactory system, Meis2 is strongly expressed in neuroblasts (labeled by TuJ1, PSA, NCAM, or Dcx) and colocalizes extensively with Pax6 in migrating neuroblasts in the subventricular zone, the rostral migratory stream, and the olfactory bulb (Pennartz et al., 2004). In contrast, it is absent from stem cells or astrocytes (GFAP+) and from transient progenitors (Ascl1+) during adult neurogenesis (Marei et al., 2012; Agoston et al., 2014). Therefore, Meis2 appears to be essential for the specification of the olfactory bulb (Brill et al., 2008), and this is likely conserved in *Xenopus*.

In the *Xenopus* pallial regions, the expression of Meis2 is evident from embryonic stages. This expression becomes progressively restricted to the ventrolateral part, and throughout the larval and adult stages, it is localized to the ventropallial region. Double labeling with telencephalic markers, such as CR, which shows the boundary with the striatum (Morona and González, 2008), or Otp, which marks amygdaloid populations (Jimenez and Moreno, 2022a,b; Lozano et al., 2025), supports the idea of an essential role in the differentiation of the pallial-subpallial border and the precise regionalization of ventropallial derivatives. In mice, Meis2 is expressed in a subclass of cortical interneurons expressing serotonin receptor 3 A (Htr3a) within the white matter. It is very rare in the gray matter and co-expresses SP8 and ER81 but not PROX1. These interneurons originate from a specific region of the pallio-subpallial border, which is characterized by Meis2+/PROX1–, outside the limits of the caudal ganglionic eminence. Interestingly, they have a strong genetic relationship with olfactory bulb interneurons, while the expression profiles of transcription factors typical of cortical interneurons derived from the caudal ganglionic eminence are low (Allen et al., 2007; Frazer et al., 2017). Meis2 not only shows differential expression at the pallial-subpallial boundary, where it is mainly involved in neuronal differentiation and axonogenesis, but it is also associated with the expression of regionalization genes at these boundaries such as Dlx1/2 and Pax6 (Su et al., 2022). Similar patterns have been observed in other types of boundaries, such as the prethalamus (Andrews et al., 2003; Alonso et al., 2020). In comparison, the expression of Meis2 in *Xenopus* is restricted to ventropallial populations, suggesting a regional reduction in the pallial structures, related either to a smaller cortical extent in *Xenopus* or to differences in the specification of cell types in these regions, which are associated with a more limited expression of genes related to its development.

The expression of Meis2 in striatal cells is a distinctive feature of Xopus development. This pattern is well conserved in mammals, where Meis2 is present from early stages and contributes to the specification of medium spiny neuron fate (Larsen et al., 2010; Su et al., 2022). In contrast, this population is largely absent in adult Xenopus, with only a few scattered Meis2-immunoreactive (Meis2-ir) cells remaining. With regard to the subpallial region, Meis2 is specifically expressed in the subventricular zone of the medial ganglionic eminence (Petryniak et al., 2007). In the adult pallidal derivatives of this region, a distinction has been described between two main populations of neurons in the lateral septum: those arising from the rostral septum, which express Meis2, and those from the caudal septum, which express Nkx2.1 (Reid et al., 2024). In addition, it has been proposed that Nkx2.1 represses Meis2 expression, a mechanism consistent with their complementary distribution (Sandberg et al., 2016). In zebrafish, it has been documented that at advanced larval stages, Meis2 expression persists in the ventrobasal telencephalon (Biemar et al., 2001; Zerucha and Prince, 2001). In the case of *Xenopus*, within the subpallial region, Meis2 expression is observed in pallidal derivatives, particularly in septal derivatives and the bed nucleus of the stria terminalis, suggesting a comparable expression pattern in this region.

Finally, in relation to the telencephalic populations described in *Xenopus*, analysis using Otp has allowed the identification of Meis-ir in amygdaloid regions. It is noteworthy that the expression of Otp in this region is a highly evolutionarily conserved feature across all vertebrates (Lozano et al., 2025).

Recently, in the human hypothalamus, mutually exclusive alar ventricular domains were detected in a complete spatio-cellular atlas based on the expression of Meis2 and OTP (Tadross et al., 2025). In the mouse hypothalamus, Meis2 has been exclusively associated with a population of Sst+ GABAergic neurons and a glutamatergic subpopulation of the lateral hypothalamic area (LHA). Specifically, Meis2 is expressed in the Sst-positive perifornical neurons of the LHA but not in Sst+ tuberal neurons, in contrast to Otp, which is present in the tuberal region (Mickelsen et al., 2019). The expression of Meis2 in the hypothalamus has also been described in advanced larval stages of zebrafish (Biemar et al., 2001; Zerucha and Prince, 2001). In *Xenopus*, the combination of Meis2 with Otp, a conserved marker of the paraventricular region involved in the development of neuroendocrine nuclei (Morales-Delgado et al., 2011), situated Meis2-ir cells in the Otp-negative subparaventricular domain, demonstrating the anatomical conservation pattern of Meis2 in the hypothalamic-telencephalic region.

In the diencephalic region, the inhibitory neurons of the thalamic reticular nucleus, which surround the mouse thalamus in a shell shape, expressed Meis2 together with other key transcription factors such as Dlx1, Dlx2, Dlx5, Dlx6, Islet1, and Pax6, originated embryologically from the prethalamic region (Puelles et al., 2021; Kim et al., 2025). In *Xenopus*, Pax6 expression in the alar p3 and calretinin labeling in the thalamus support the precise delimitation of the prethalamus (Morona and González, 2008; Morona et al., 2020) and locate Meis2 expression in the zona incerta and ventro-rostral prethalamic nuclei in the adult *Xenopus*. Both nuclei exhibit the same markers as the thalamic reticular nucleus in mammals (Bachy et al., 2002; Brox et al., 2003; Moreno et al., 2008a,d), suggesting a common molecular identity of Meis2-expressing p3-derived populations in mammals and amphibians.

Meis2 has been described as essential for midbrain development through several mechanisms in mammals. Firstly, it is involved in tectal fate specification, permitting the activation of Otx2 even in the absence of the isthmic organizer (Agoston and Schulte, 2009). Furthermore, it triggers additional downstream genes of Otx2, such as Pax3 and Pax7, to specify tectal fate (Agoston et al., 2012). Finally, it actively contributes to the regulation of ephA8 through a constitutive interaction essential for its activation in a rostro-caudal gradient in the developing superior colliculus, contributing to the proper establishment of the retino-collicular projection that suggests a functional implication in the regional specification of the visual pathways (Shim et al., 2007). The comparative expression pattern of Meis2 along the optic tectum and torus in *Xenopus* and mammals supports the evolutionary conservation of Meis2 and its associated network in the specification and differentiation of structures involved in visual processing. This strengthens the idea that the molecular mechanisms regulating dorsal midbrain organization and retino-collicular connectivity are widely conserved in vertebrates. Regarding the visual system, Meis2 (together with Meis1) also plays an important role in retinal development, contributing to the maintenance of the progenitor cell population by directly promoting Pax6 expression (Zhang et al., 2002) and simultaneously restricting the activation of genes associated with the ciliary margin and optic disc (Dupacova et al., 2021; Antosova et al., 2016).

In mice, Meis2 expression in the cerebellum has been implicated in the specification and development of cerebellar subpopulations in relation to the nuclear transient zone (NTZ). Two Meis2+ populations appear during development: a minority population derived from the caudodorsal rhombic lip and a main population derived from the rostroventral region, which co-expresses Otx2 and p75ntr, is Atoh-negative, and may be of mesencephalic origin (Ghiyamihoor et al., 2025). This rostral Meis2 population follows a migration route from the midbrain to the NTZ, developing cerebellar nuclei in an atypical path (Rahimi-Balaei et al., 2024). In *Xenopus*, during early embryonic stages, Meis2-positive cells are not detected in the prospective cerebellum or rhombic lip domains, preventing a direct comparison with early-born Meis2 mammalian cerebellar cells. However, at later stages, Meis2ir has been identified in cerebellar populations outside the cerebellar lamina, which is defined by calbindin immunoreactivity in the Purkinje cell layer and Pax6 in the granular population (Bandin et al., 2014). Meis2-ir cells appeared both in rostral regions near the mesencephalon and in more caudal areas. This pattern suggests a conserved transcriptional program in cerebellar specification, where Meis2 may play a role in the specification of regions related to the nuclear zones described in mammals.

In the hindbrain, Meis2 is notable for its intense expression near the mid-hindbrain boundary, where it appears independently of the appearance of Fgf8 in the organizer (Vennemann et al., 2008). In the rostral hindbrain, Meis2 acts synergistically with the Hox/Pbx complex (Vitobello et al., 2011) to initiate Krox20 expression in the r3 segment (Wassef et al., 2008). In addition to the direct transcriptional control of Meis2 in segmentation and the specification of rhombomeric identity, other members of the family, such as Meis1, have been proposed as candidates for specification maintenance at later stages, suggesting possible functional redundancy or temporal progression in their regulation (Vennemann et al., 2008; Wassef et al., 2008). These studies were carried out in chicken and mouse embryos, but the molecular mechanisms of rhombencephalic segmentation, especially those related to Hox genes, are highly conserved in vertebrates (Krumlauf et al., 1993; Parker and Krumlauf, 2020). This indicates that the function of Meis2 in r3 and the regulation of Krox20 probably also occur in *Xenopus* or Zebrafish. Precisely, the early co-localization of Meis2 with Otx2 in zebrafish confirmed that Meis2 expression is initially confined to the presumptive rhombencephalon (Biemar et al., 2001; Zerucha and Prince, 2001). As somitogenesis progresses, Meis2 is distributed in the anterior rhombomeres (r1-r3), and, at more advanced stages, intense expression appears in rhombomeres r3 and r4 (Biemar et al., 2001; Zerucha and Prince, 2001). These results are consistent with those found in *Xenopus* development, maintaining a conserved pattern.

## 5 Conclusion

In *Xenopus laevis* development, Meis2 expression in the telencephalon is restricted to ventropallial regions from embryonic to adult stages, as well as to pallidal derivatives, such as septal groups and the bed nucleus of the stria terminalis. Nevertheless, within the striatum, it is barely observed, in clear contrast to its prominent presence in mammals. This discrepancy may reflect evolutionary differences in cell specification or the lack of certain cell types in the striatum of *Xenopus*. In the hypothalamus, Meis2 is expressed in the subparaventricular domain, maintaining a pattern similar to that observed in zebrafish and mammals. In the midbrain, Meis2 expression in the optic tectum of Xenopus and the superior colliculus of mice suggests strong conservation of the molecular program involved in the development of the visual system. In the cerebellum, Meis2 is detected in regions equivalent to the cerebellar nuclei, reflecting a pattern and developmental origin shared with mammals in the specialization of distinct cerebellar populations. Finally, in the hindbrain, Meis2 is expressed early in the anterior rhombomeres (r1–r3), as observed in zebrafish and mice, and soon spreads throughout the entire rhombencephalon. Consequently, Meis2 exhibits a substantial degree of evolutionary conservation in vertebrates, integrating regional patterning, neuronal differentiation, and circuit assembly through conserved molecular interactions. Specifically, the significant preservation of Meis2 in the hindbrain underscores its critical role in neuronal development and its potential as a therapeutic target for neurodevelopmental pathologies. The divergences described can be mostly attributable to the mechanisms of cell specification associated with species-specific evolutionary changes. Future studies should explore these cell type-specific mechanisms and evolutionary adaptations.

## Data Availability

The original contributions presented in the study are included in the article/Supplementary material, further inquiries can be directed to the corresponding author.

## Supplementary material

Supplementary Figure 1 Table of developmental stages of *Xenopus laevis* summarized after Nieuwkoop and Faber (1967) and the numbers of experimental animals used in each stage.

Supplementary Figure 2 Schematic diagrams of the rostrocaudal distribution of Meis2-ir in the brain of early embryonic stages. Dots represent the location of Meis2ir cells. In all schemes dorsal is oriented upward. The main subdivisions are indicated by dashed lines. The approximate levels are indicated in the upper sagittal scheme. For abbreviations, see list.

Supplementary Figure 3 Schematic diagrams of the rostrocaudal distribution of Meis2-ir in the brain of late embryonic stage. Dots represent the location of Meis2-ir cells. In all schemes dorsal is oriented upward. The main subdivisions are indicated by dashed lines. The approximate levels are indicated in the upper sagittal scheme. For abbreviations, see list.

Supplementary Figure 4 Schematic diagrams of the rostrocaudal distribution of Meis2-ir in the brain of premetamorophic larval stage. Dots represent the location of Meis2-ir cells. In all schemes dorsal is oriented upward. The main subdivisions are indicated by dashed lines. The approximate levels are indicated in the upper sagittal scheme. For abbreviations, see list.

Supplementary Figure 5 Schematic diagrams of the rostrocaudal distribution of Meis2-ir in the brain of prometamorophic larval stage. Dots represent the location of Meis2-ir cells. In all schemes dorsal is oriented upward. The main subdivisions are indicated by dashed lines. The approximate levels are indicated in the upper sagittal scheme. For abbreviations, see list.

Supplementary Figure 6 Schematic diagrams of the rostrocaudal distribution of Meis-2ir in the brain of metamorphic climax stages. Dots represent the location of Meis2-ir cells. In all schemes dorsal is oriented upward. The main subdivisions are indicated by dashed lines. The approximate levels are indicated in the upper sagittal scheme. For abbreviations, see list.

## Glossary

ABB, alar-basal boundary; Ad, anterodorsal tegmental nucleus; AOB, accessory olfactory bulb; Av, anteroventral tegmental nucleus; BST, bed nucleus of the stria terminalis; Cb, cerebellum; cLLN, caudal nucleus of the lateral line; CoP, commissural pretectal domain; d, dorsal; DCN, dorsal column nucleus; DP, dorsal pallium; gl, glomerular layer of the olfactory bulb; GT, griseum tectale; H, hypothalamus; Hb, habenula; Igl, internal granular layer of the olfactory bulb; Ip, interpeduncular nucleus; Is, isthmic nucleus; LA, lateral amygdala; Lc, locus ceruleus; LC, laterocaudal mesencephalic nucleus; LDT, laterodorsal tegmental nucleus; LLN, lateral line nucleus; LP, lateral pallium; LR, laterorostral mesencephalic nucleusLR; Ma, mamillary nucleus; MeA, medial amygdala; Mes, mesencephalon; MGE, medial ganglionic eminence; ml, mitral layer; MP, medial pallium; nCb, cerebellar nucleus; Nsol, nucleus of the solitary tract; OB, olfactory bulb; oc, optic chiasm; Oi, inferior olivary complex; Ols, superior olivary complex; OPT, olivary pretectal nucleus; OT, optic tectum; p1-p3, prosomeres 1 to 3; Pa, paraventricular hypothalamic domain; pA, pallial amygdala; IC, intercalate thalamic nucleus; Pal, pallium; Pb, parabrachial area; pc, posterior commissure; Pdi, posterodorsal isthmic nucleus; PO, preoptic area; PPT, principal pretectal nucleus; r, rostral; r0, isthmus; r1-r8, rhombomeres 1 to 8; Ra, raphe; Ras, superior raphe column; Rh, rombencephalon; Ri, inferior reticular nucleus; Ris, isthmic reticular nucleus; rLLN, rostral nucleus of the lateral line; Rm, medius reticular nucleus; Rs, superior reticular nucleus; S, septum; Sd, dorsal septum; sg, stratum granulare of the OT; sgv, stratum granulare ventriculare of the OT; Sl, lateral septum; Sm, medial septum; Sol, solitary tract; SPa, subparaventricular hypothalamic domain; Spal, Subpallium; St, stadio; St, striatum; Tegm, mesencephalic tegmentum; Tel, telencephalon; Tmg, torus magnocellularis; Tp, torus principalis; Tu, tuberal hypothalamic domain; v, ventricle; Vc, ventrocaudal prethalamic nucleus; VP, ventral pallium; Vpr, principal sensory trigeminal nucleus; Vr, ventro-rostral prethalamic nucleus; VTA, ventral tegmental area; ZI, zona incerta.
