# Supplementary figures and images for: Developmental and adult expression of the Meis2 transcription factor in the central nervous system of Xenopus laevis: a developmental and evolutive analysis
Source: Front Neuroanat. 2025 Nov 6;19:1677413. doi: 10.3389/fnana.2025.1677413 (PMC12631407; doi:10.3389/fnana.2025.1677413)

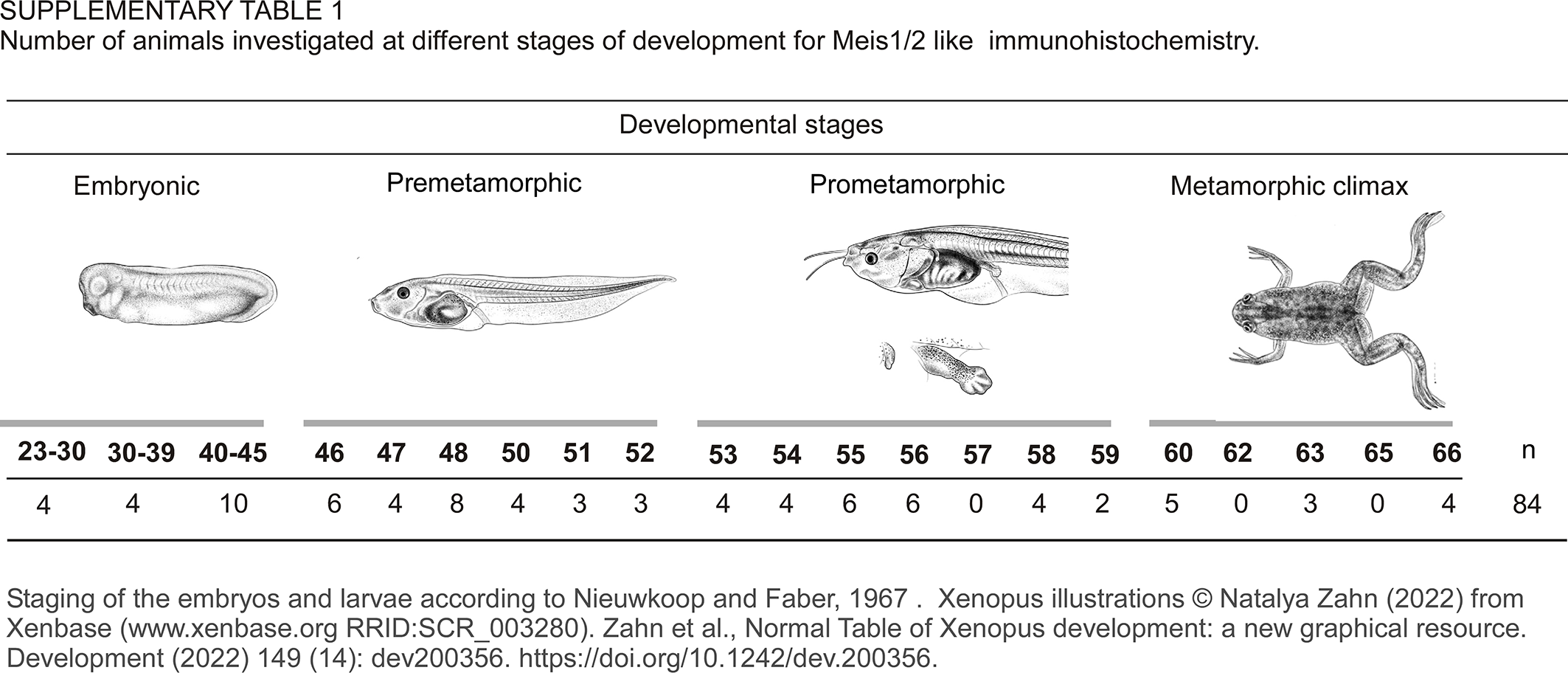

Supplement: Supplementary Figure 1 — Table of developmental stages of Xenopus laevis summarized after Nieuwkoop and Faber (1967) and the numbers of experimental animals used in each stage. [file Data_Sheet_1.zip › Suplementarias R01/Sup 1.tif]

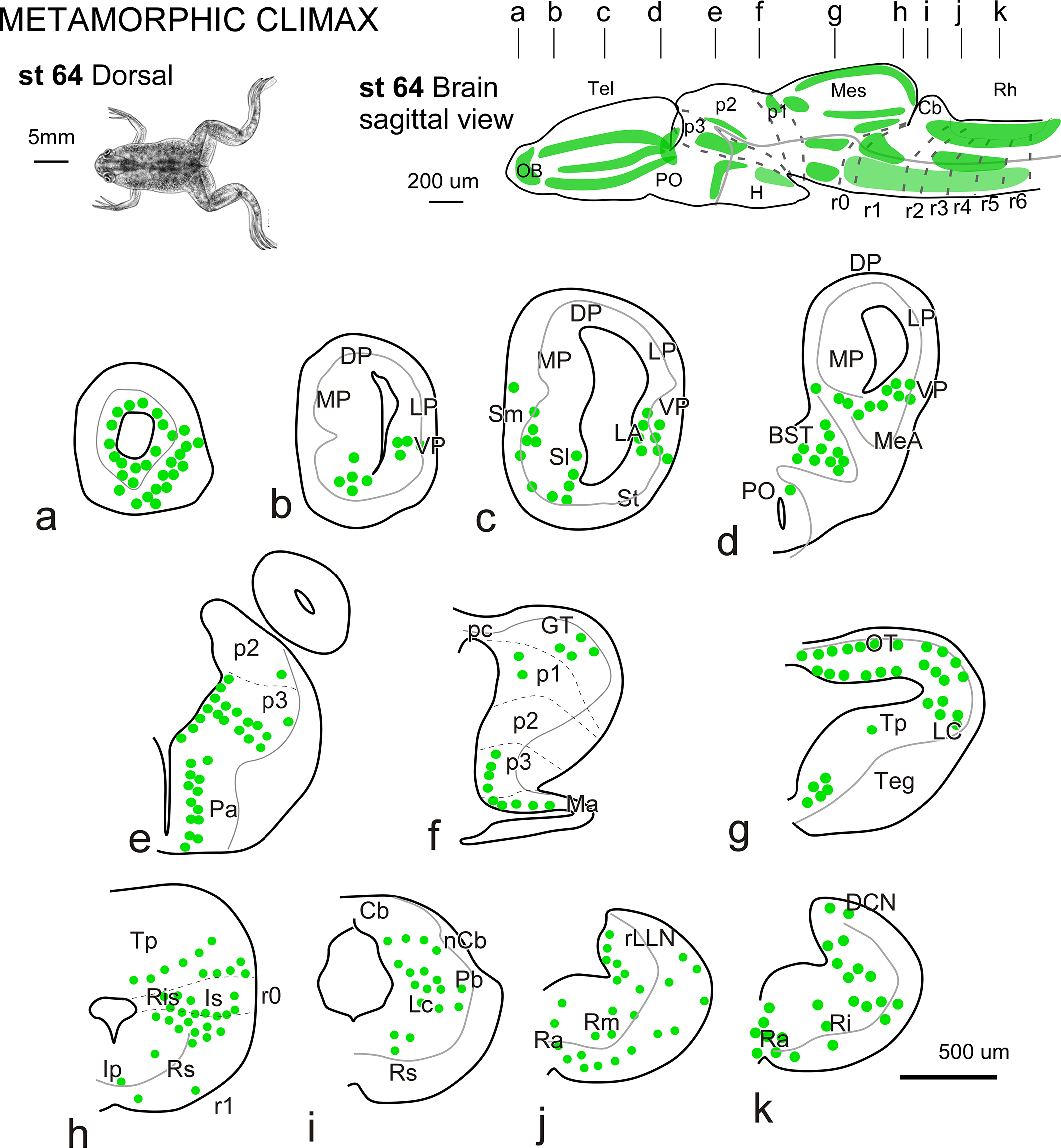

Supplement: Supplementary Figure 1 — Table of developmental stages of Xenopus laevis summarized after Nieuwkoop and Faber (1967) and the numbers of experimental animals used in each stage. [file Data_Sheet_1.zip › Suplementarias R01/Sup 6.tif]

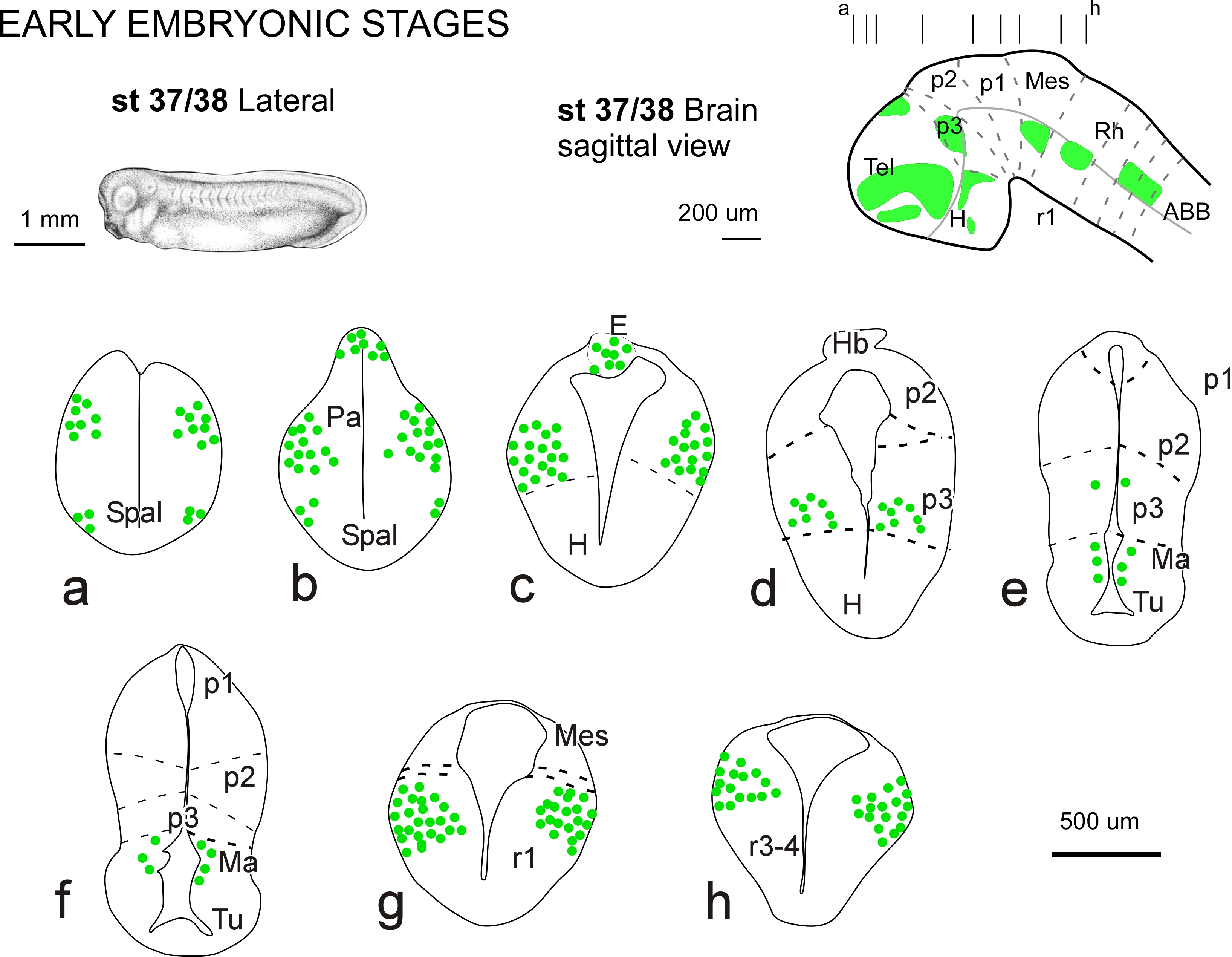

Supplement: Supplementary Figure 1 — Table of developmental stages of Xenopus laevis summarized after Nieuwkoop and Faber (1967) and the numbers of experimental animals used in each stage. [file Data_Sheet_1.zip › Suplementarias R01/Sup 2.jpg]

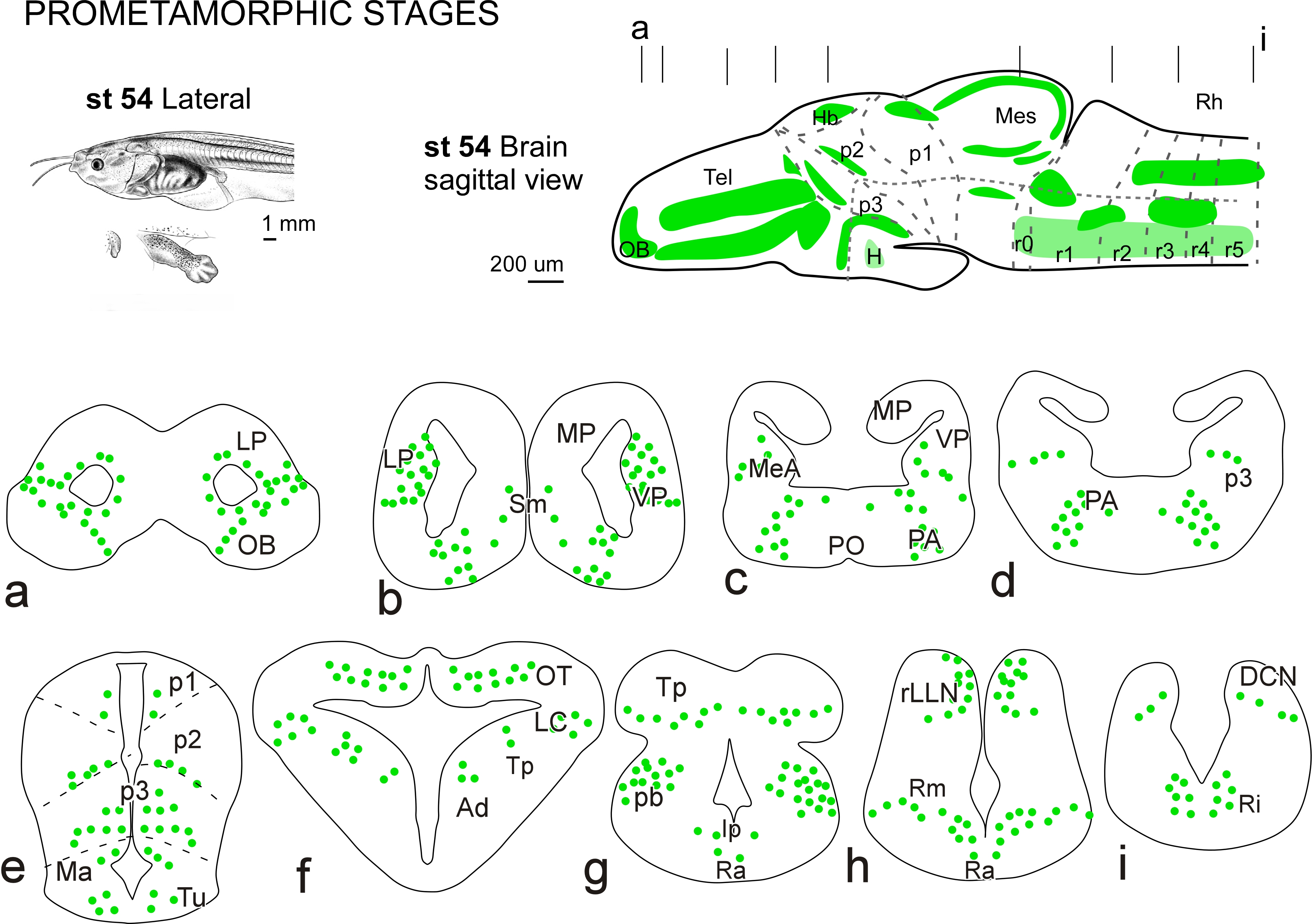

Supplement: Supplementary Figure 1 — Table of developmental stages of Xenopus laevis summarized after Nieuwkoop and Faber (1967) and the numbers of experimental animals used in each stage. [file Data_Sheet_1.zip › Suplementarias R01/Sup 5.jpg]

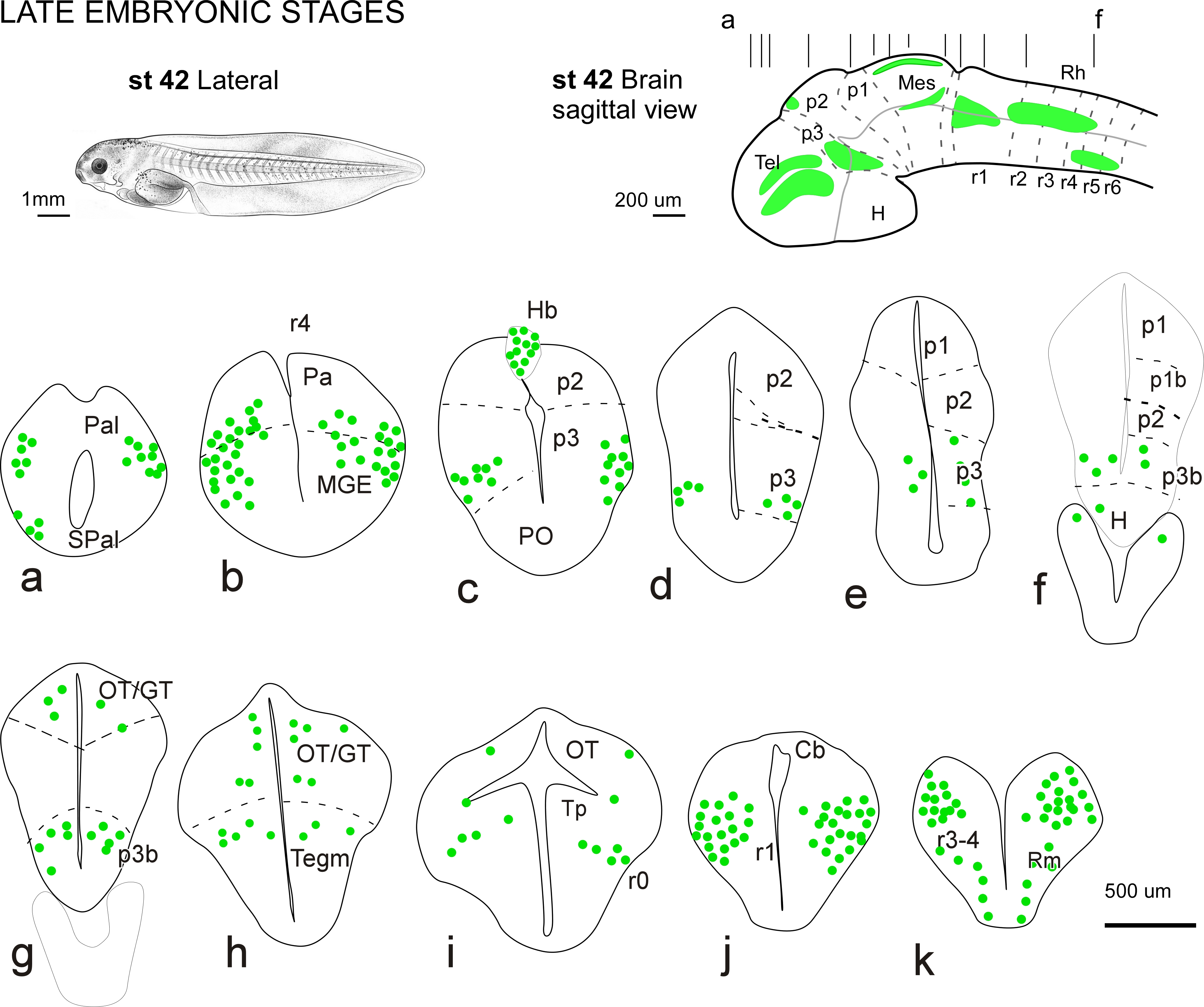

Supplement: Supplementary Figure 1 — Table of developmental stages of Xenopus laevis summarized after Nieuwkoop and Faber (1967) and the numbers of experimental animals used in each stage. [file Data_Sheet_1.zip › Suplementarias R01/Sup 3.jpg]

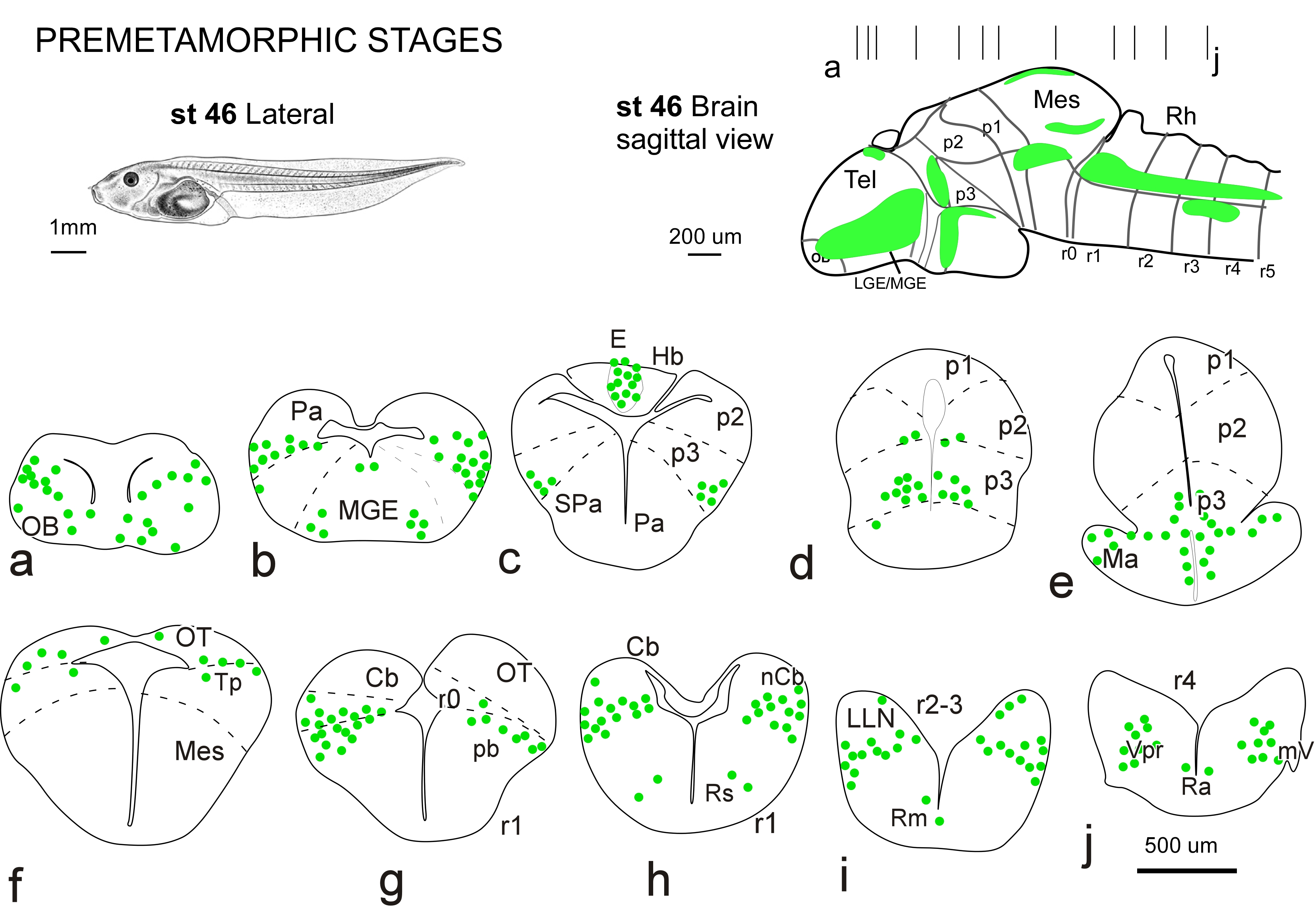

Supplement: Supplementary Figure 1 — Table of developmental stages of Xenopus laevis summarized after Nieuwkoop and Faber (1967) and the numbers of experimental animals used in each stage. [file Data_Sheet_1.zip › Suplementarias R01/Sup 4.jpg]
